# Major Evolutionary Trends in Hydrogen Isotope Fractionation of Vascular Plant Leaf Waxes

**DOI:** 10.1371/journal.pone.0112610

**Published:** 2014-11-17

**Authors:** Li Gao, Erika J. Edwards, Yongbo Zeng, Yongsong Huang

**Affiliations:** 1 Department of Earth, Environmental and Planetary Sciences, Brown University, Providence, Rhode Island, United States of America; 2 Department of Ecology and Evolutionary Biology, Brown University, Providence, Rhode Island, United States of America; 3 Department of Electrical, Computer, and Biomedical Engineering, University of Rhode Island, Kingston, Rhode Island, United States of America; Estación Experimental del Zaidín (CSIC), Spain

## Abstract

Hydrogen isotopic ratios of terrestrial plant leaf waxes (δD) have been widely used for paleoclimate reconstructions. However, underlying controls for the observed large variations in leaf wax δD values in different terrestrial vascular plants are still poorly understood, hampering quantitative paleoclimate interpretation. Here we report plant leaf wax and source water δD values from 102 plant species grown in a common environment (New York Botanic Garden), chosen to represent all the major lineages of terrestrial vascular plants and multiple origins of common plant growth forms. We found that leaf wax hydrogen isotope fractionation relative to plant source water is best explained by membership in particular lineages, rather than by growth forms as previously suggested. Monocots, and in particular one clade of grasses, display consistently greater hydrogen isotopic fractionation than all other vascular plants, whereas lycopods, representing the earlier-diverging vascular plant lineage, display the smallest fractionation. Data from greenhouse experiments and field samples suggest that the changing leaf wax hydrogen isotopic fractionation in different terrestrial vascular plants may be related to different strategies in allocating photosynthetic substrates for metabolic and biosynthetic functions, and potential leaf water isotopic differences.

## Introduction

Hydrogen isotopic ratios (δD) of vascular plant leaf waxes are widely used for paleoclimate reconstruction from geological archives [1]–[9]. Leaf wax δD values preserved in lake and ocean sediments provide a record of precipitation isotopic ratios, allowing reconstruction of changes in hydrology and temperature for time scales of thousands to millions of years. However, hydrogen isotopic fractionation of terrestrial vascular leaf waxes is highly variable, differing by up to 80 ‰ across species [10]–[13], and the underlying controls for this variability are poorly understood, hampering quantitative paleoclimate interpretation.

Variations in vascular plant leaf wax hydrogen isotopic fractionation have been tentatively linked to growth forms [10]–[13]. For example, grasses typically have 30 to 50 ‰ lower δD values than trees grown in a similar environment [10]–[12]. Such grouping is, however, potentially problematic because growth forms are simple visual characteristics and generally do not correspond to fundamental biochemical and physiological processes, and perceived statistical differences when data are grouped by plant functional type can be misleading [14]. Published data also contain plants collected from very different geographic locations with large differences in precipitation isotopic ratios, climatic and environmental conditions [13]. Differing ambient temperature, relative humidity, soil type and other environmental factors could also potentially affect the isotopic fractionation. Most of the published fractionation values are calculated relative to modeled δD values at different localities [13], [15], introducing added uncertainties. Because samples are collected based on morphological classification, there is considerable sampling bias in terms of plant phylogeny: more than 95% of the previously reported plant species are from only two major plant lineages, the eudicots and Poales [13] (Fig. S1), making general inferences across all plants difficult.

To avoid both environmental heterogeneity and poor taxonomic representation, we acquired comprehensive isotope data for plant leaf waxes, irrigation and xylem waters from 102 species from New York Botanic Garden (Table S1), and performed phylogenetically-informed analyses to critically examine the potential effects of plant types on the leaf wax hydrogen isotopic fractionation. Our objectives are thus: 1) to produce leaf wax hydrogen isotopic fractionation data with careful constraints on plant source water and minimal variability in growth environments, and 2) to explore the potential influences of plant phylogeny and other plant characteristics (e.g., growth habits, photosynthetic pathways) on vascular plant leaf wax hydrogen isotope fractionation.

## Materials and Methods

### Samples from the New York Botanic Garden

All the 102 terrestrial vascular plant species were collected from the New York Botanic Garden (NYBG: Coordinates: 40.8636° N, 73.8783° W) in the USA (Table S1) following our standard lab procedures [12],[16]. The majority of the samples were collected on May 28, 2009. Additional ferns were collected on July 10, 2010 (marked with * in Table S1). The lycopods were collected on Aug 15, 2009. All the samples were collected between 12pm and 2 pm. Fresh leaves for leaf wax analysis were stored in whirl-pak bags, while those for leaf water δD analysis were immediately sealed in glass vials. Stems (wherever available) were collected to measure the xylem water δD. The New York Botanical Garden issued the permit for sampling the 102 samples from the NYBG (Contact: Mr Jon Peter). The different sampling times were due to constraints on field trip timing, and required supplementary samples as the project progressed. Because we simultaneous determined xylem water δD values, we believe the influence of variable sampling times on our calculated isotopic fractionation values should be small and negligible.

### Field samples from Blood Pond, Massachusetts and growth chamber experiments

We also report additional data from field samples and growth chamber experiments, aimed at better understanding the hydrogen isotopic differences between grasses and dicots. We collected leaf materials for leaf wax and leaf water δD analysis for selected grasses and trees around Blood Pond (42.0814°N, 71.9618°W, 212.1 m a.s.l.), Massachusetts at different stages of the growth season (May, June, September in 2007), following the same protocol as reported in Gao et al. [12]. The sampling dates and data are given in Table S2. We followed the procedures of growth chamber experiments closely as described in Hou et al. [17]. We grew 19 trees (representing 5 species) and 19 grasses (representing 3 species) in a GC series temperature and humidity-controlled growth chamber by Environmental Growth Chamber Company (Data as shown in Table S2). The temperature in the growth chamber was kept at 20°C. RH was kept constant at 80% during the growing period (Jun 26–Jul 27, 2006). Blood Pond (Coordinates: 42.0814°N, 71.9618°W, 212.1 m a.s.l.) is private land for the Koebke Farm, who should be contacted for further permissions. The growth chamber experiments were performed at the Greenhouse of Brown University (Contact: Prof. Fred Jackson), and no specific permissions are required. The field studies did not involve endangered or protected species.

### Analytical methods for leaf waxes

Sample preparation, and leaf wax, leaf water and xylem water δD analysis was conducted following the same procedure as in Gao et al. [16], [18]. Briefly, leaf lipids were extracted from freeze-dried leaves and quantified using a Hewlett-Packard 6890 gas chromatograph (GC) with split/splitless injector and a flame ionization detector (FID); compound identification was based on retention times of lipid standards on an Agilent 6890N GC coupled to an Agilent 5973N quadrupole mass analyzer. Data of leaf wax compositional information are presented in files Fig. S2, Table S3 and Table S4 (SI is supporting information). D/H ratio analysis was carried out on an HP 6890 GC, interfaced to a Finnigan MAT Delta+ XL isotope ratio mass spectrometer (IRMS) via a high-temperature pyrolysis reactor. The H^3+^ factor was 2.6±0.3 during the analysis and analytical errors were <2.5 ‰ for IRMS based on repeated analysis of our laboratory standards (mixture of *n*-C_22_, -C_24_, -C_26_, -C_28_ and -C_30_ fatty acid methyl esters; and *n*-C_25_, -C_27_, -C_29_, -C_30_, and -C_32_ alkanes). The plant samples were analyzed in duplicates or triplicates to ensure repeatability (with analytical error less than 2.5 ‰ for the majority of the samples). Leaf and xylem water was distilled using the cryogenic system at Brown University and water δD was measured on a Picarro L1102-i isotopic liquid water and water vapor analyzer (Sunnyvale, CA, USA) following procedures described in Gao et al. [16]. The standard deviation for repeated standard analysis was <0.1 ‰ for δ^18^O and <0.6 ‰ for δD during our measurements, and the machine was monitored continuously with a lab standard (−38 ‰ δD) for every 9 samples. The irrigation water used for growth experiments had a δD of −49 ‰.

### Xylem water and the environment water

We measured xylem water hydrogen isotopic ratios for the majority of plant samples except for 15 ferns and 10 lycopods (Table S1). These fern and lycopod samples have green, photosynthetically-active stems which contain isotopically enriched xylem water due to evapotranspiration. These plants are cultivated in moist and shaded environments with regular irrigation water at NYBG, which minimize soil water evaporation. Thus, the annual mean precipitation water δD (−57 ‰) was used to calculate the fractionation of these samples. This value was obtained from the Online Precipitation Isotopes Calculator (http://wateriso.utah.edu/waterisotopes/pages/data_access/oipc.html).

### Calculation of hydrogen isotope fractionation between leaf lipids and water

The raw isotopic data of the NYBG samples include δD values of leaf waxes (*n*-C_24_, C_26_, C_28_ and C_30_ alkanoic acids and *n*-C_27_, C_29_, and C_31_ alkanes), δD of xylem water (δD_xylem_) and δD of environmental water (δD_environ_, −57 ‰; Table S1). The hydrogen isotope fractionation between leaf waxes and environmental water (ε_wax-environ_) was calculated by the equation ε_wax-environ_  =  ((δD_wax_+1)/(δD_environ_+1)-1, where δD_wax_ is the δD value of each of the above individual lipids. Similarly, the hydrogen isotope fractionation between leaf waxes and xylem water (ε_wax-xylem_) was calculated by the equation ε_wax-xylem_  =  (δD_wax_+1)/(δD_xylem_+1)-1.

### Integrating compound-specific δD values of leaf waxes

**Figure 1:**
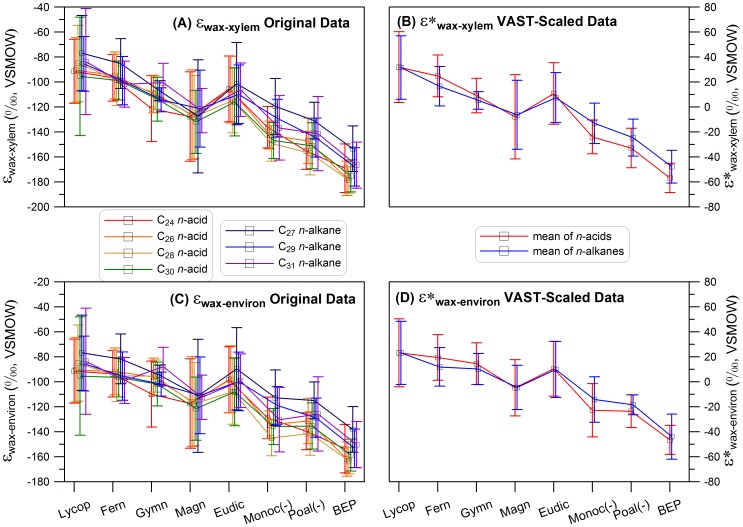
Leaf wax hydrogen isotope fractionation relative to environmental water (ε_wax-environ._) and xylem water (stem water, ε_wax-xylem_) for all major lineages of terrestrial vascular plants. Lycop  =  Lycopod; Gymn  =  Gymnosperm; Magn  =  Magnoliids; Eudic  =  Eudicots; Monoc(-)  =  Monocots excluding Poales; and Poal(-). =  Poales excluding BHP; BEP comprises subfamilies Bambusoideae, Ehrhartoideae and Pooideae within the graminoids. The error bars show the 1 σ standard deviation for all the species in individual lineages. A and C are for original data of ε_wax-environ._ and ε_wax-xylem_ values. B and D are the scaled and averaged data for the two sets of values (ε*_wax-environ._ and ε*_wax-xylem_), respectively. See supplementary information on the rationale and methods for scaling the isotopic measurements.

To reveal the hydrogen isotope fractionation variations among major phylogenetic lineages, we calculated the average and standard deviation values for each lineage for individual lipids (Figs. 1A and 1C). These figures show that variations of ε_wax-xylem_ and ε_wax-environ_ values for individual lipids among different phylogenetic lineages follow similar trends. Therefore, isotopic variation in any single lipid is in general representative of the overall differences among different plant lineages. We further calculated the correlation of ε_wax-xylem_ values between different leaf lipids for all plants and found the lipid ε_wax-xylem_ values are positively correlated (Fig. S3). Such positive correlation also exists for ε_wax-environ_ values in our data (figures not shown), and has been reported previously [10]–[12], [19].

Therefore, the best approach to compare the isotopic differences between different plant lineages is to obtain an integrated isotopic value from the measurement of seven individual compounds (i.e., *n*-C_24_, C_26_, C_28_ and C_30_ alkanoic acids and *n*-C_27_, C_29_, and C_31_ alkanes). There are a number of advantages in obtaining an integrated isotopic value over relying on individual compounds for plant comparison: 1) integration takes advantage of multiple measurements of different compounds rather than individual compound for a given plant sample, reducing the potential influence of outliers and enhancing the likelihood of obtaining the most representative isotopic data; 2) occasionally, concentrations for certain individual compounds from a plant sample are too low or contain coeluting compounds on GC-IRMS for accurate isotopic measurements, so an integration from available measurements avoids data gaps; and 3) a greater number of isotopic observations, by considering all compounds for a single plant provides a more statistically robust representation of isotopic fractionation in certain plants.

However, we cannot simply take the mathematical means of δD values of different compounds in a given sample to obtain an integrated isotopic mean value, because there are some natural offsets in hydrogen isotopic values between different compound classes (e.g., alkanes, acids) and between different carbon chain length compounds of the same compound classes. For example, ε values between C_28_ *n*-acid and environmental water for all plants are positively correlated to those between C_30_ *n*-acid and environmental water (R^2^ = 0.78), but the correlation is not 1∶1 and the interception is not zero (Fig. S3).

To obtain integrated values for single plants, we followed the well-established statistical procedure described as follows. We first centered the ε_wax-xylem_ values and ε_wax-environ_ values for each lipid by subtraction of the mean values for each lipid. We then scaled the centered values using the variable stability (VAST) scaling method [20]. This method weights each variable according to a matrix of its stability and improves the class distinction. The weights were determined by
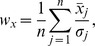
where *x_j_* and *σ_j_* denote the mean and standard deviation of a variable *x* for the *j* ^th^ class, respectively, and *n* is the total number of classes. In this way prior class information is incorporated into VAST scaling. This method is comparable to the scaling in a block fashion, utilizing the most appropriate scaling method for each variable group, particularly if different types of variables are combined [21]–[22]. We have further divided each data point by the average of all the values. By centering and scaling, all variables (7 lipids) can be treated equally to represent the plant (Table S5).

Using the centered and VAST scaled data, we obtained the ε*_wax-xylem_ values of 4 individual *n*-acids and 3 individual *n*-alkanes for each plant (Table S5). We then treated *n*-acids and *n*-alkanes separately and further obtained the average values of the 4 *n*-acids (ε*_wax-xylem-acid_) and the average value of the 3 *n*-alkanes (ε*_wax-xylem-alkane_), respectively_._ Treating *n*-acids and *n*-alkanes separately is mainly based on a Principle Component Analysis of the individual δD values of the 7 leaf lipids from each plant, suggesting *n*-acids and *n*-alkanes were separated by the 2^nd^ major component (Fig. S4). We used the two combined values as two separate entries to represent one individual plant. We also calculated the averages and standard deviations of all entries from each lineage for both *n*-acids and *n*-alkanes (Fig. 1B). We then performed a standard analysis of variance (ANOVA) of these values among different lineages to test if the group means are equal to each other. We then performed the post hoc analysis of these values and evaluate how each two group means are different. By incorporating all available data, we take full advantage of our extensive isotopic measurements of the seven leaf wax compounds for any given plant specimen, and reduce the uncertainty associated with single compound measurements. This method allows us to focus on comparing isotopic fractionation difference between *different plants* (which is the main purpose of this paper), without being affected by the different isotopic fractionation of *different leaf waxes in the same plants*. We treated the ε*_wax-environ_ values in the same manner (Fig. 1D).

### Phylogenetic analysis

**Figure 2:**
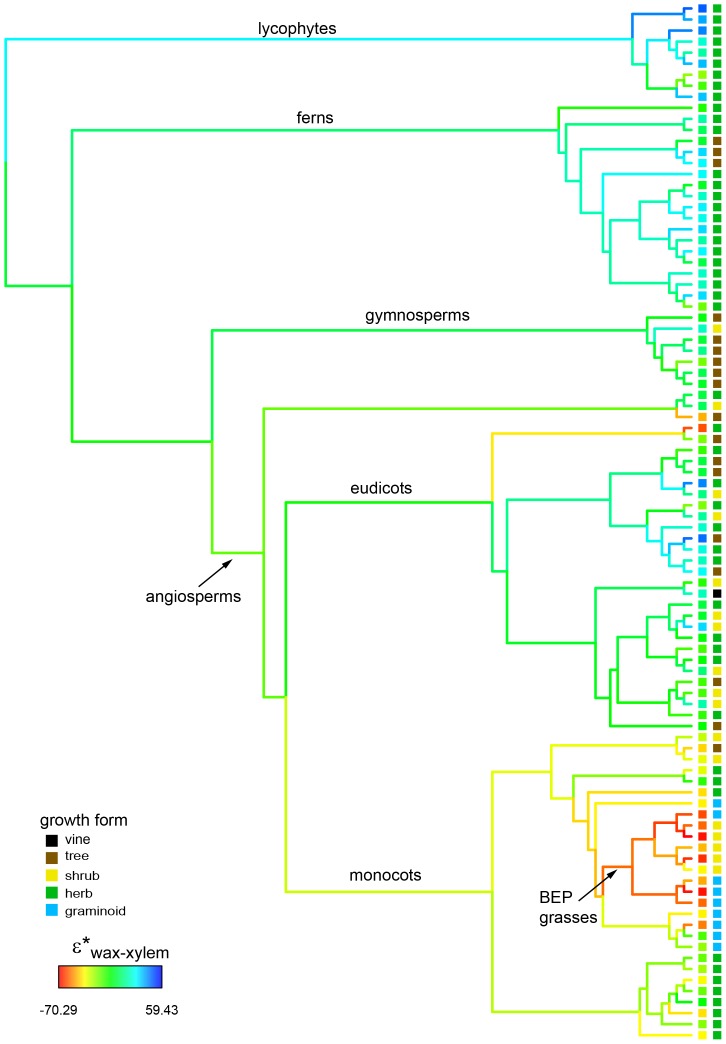
Phylogenetic patterns of mean leaf wax hydrogen isotope fractionation values of *n*-alkanoic acids. The tip values are the combined scaled values based on measurements of collected modern species (as in Fig. 1). The left of the two columns represent the living species values of ε*_wax-xylem-acid_, and colored branches represent inferred ancestral values. The right column represents different growth forms.

The combined *n*-acid or *n*-alkane entry for each plant was used for phylogentic analysis (Fig. 2; Fig. S5). We inferred the phylogenetic relationships of our sampled plants using the program *Phylomatic v3* (phylodiversity.net/phylomatic), and incorporated two sets of branch lengths into our analyses: branch lengths all equal to one, and branch lengths as proportional to time, adjusted with the ‘bladj’ command in the program Phylocom v4.2 [23]. We tested for phylogenetic signal in fractionation using Pagel's Lambda [24], implemented in the ‘phylosig’ function of the R module ‘phytools’ [25], with significance of the metrics tested using 1,000 simulations. We ran these analyses across both sets of branch lengths and with *ln*-transformed and untransformed datasets; in all cases we inferred significant phylogenetic signal in our hydrogen isotope fractionation data.

We also modeled the evolution of ε*_wax-xylem-acid_, comparing the relative fit of different models using AIC (Aikaike Information Criterion) scores. We fit our data to a pure Brownian motion model of trait evolution, as well as an Ornstein-Uhlenbeck stabilizing selection model, which allows for different regions of the tree to be evolving under different trait optima (Table 1). We tested several different O-U models after visually inspecting the patterns of ε*_wax-xylem-acid_ variation across our phylogeny. First, we employed a simple O-U model that selects a single trait optimum for all taxa. Second, we modeled a two-optimum scenario, with monocots evolving under a different optimum than all other vascular plants. Finally, we designed a three-optimum scenario, where BEP grasses, all non-BEP monocots, and all non-monocot plants each were evolving under distinct optima. All phylogenetic analyses were performed in R, using the packages ‘phytools’ [25], ‘ape’ [26], and ‘ouch’ [27].

**Table 1:** Evolutionary models of leaf wax hydrogen isotopic discrimination.

Model	-lnL	AIC	q	q_monocot_	q_BEP_
Brownian motion	−459.78	923.70	NA	NA	NA
OU: global optimum	−438.96	884.19	11.84	NA	NA
OU2: monocot vs all other	−422.69	853.82	16.31	−30.75	NA
OU3: BEP vs other monocot vs all other	−418.96	848.60	16.31	−27.82	−56.44

q =  modeled ‘optimum’ trait value under a stabilizing selection evolutionary model. AIC =  Aikaike Information Criterion scores accounting for sample size. L =  Likelihood scores. OU =  the Ornstein-Uhlenbeck stabilizing selection model.

## Results

### Phylogenetic patterns in hydrogen isotopic fractionation

We first grouped hydrogen isotopic fractionation data between leaf waxes and source water (the annual precipitation (−57‰) at the NYBG and the xylem water of plants) according to major phylogenetic lineages, separating taxa into lycopods, ferns, gymnosperms, magnoliids, eudicots, and monocots (Fig. 1). In monocots, we have further divided the whole lineage into three sub-categories: the monocots excluding any Poales (labeled as “Monoc (-)”), the Poales excluding the BEP clade, a lineage of exclusively C_3_ grasses including the bamboos, rice, and the cold-adapted Pooideae (labeled as “Poal(-)”) and the BEP clade (Fig. 1; Table S1; Table S5). We also examined all the sub lineages within eudicots, but found no obvious difference in ε* (thus no display in Fig 1). The fractionation computed relative to both mean annual precipitation at NYBG (used as ‘environment water’) and to xylem water shows similar trends, but the standard deviations for individual lineages are reduced by ∼20% when xylem water is used for calculation. This is not surprising, as xylem water represents actual water transported by the plant vascular system for biosynthesis.

ANOVA analysis of hydrogen isotope fractionation demonstrates statistically significant difference among several major phylogenetic lineages (Table 2). Specifically, we find that plants representing earlier-diverging lineages (e.g., lycopods, ferns) display smaller fractionation than later-diverging lineages (Fig. 1). A similar pattern also exists in the compiled published data [10], [13] when grouped by major lineage membership (Fig. S6), but with much larger standard deviations, probably reflecting uncertainties in source water isotopic ratios.

**Table 2:** ANOVA and post hoc analysis of leaf wax hydrogen isotope fractionation relative to xylem water (centered and VAST-scaled for both *n*-acids and *n*-alkanes).

ε*_wax-xylem-acid_	Lycop	Fern	Gymn	Magn	Eudic	Monoc(-)	Poal(-)
Fern	**0.87**						
Gymn	0.01	0.04					
Magn	0.00	0.00	**0.50**				
Eudic	0.00	0.00	**1.00**	**0.13**			
Monoc(-)	0.00	0.00	0.00	**0.33**	0.00		
Poal(-)	0.00	0.00	0.00	0.04	0.00	**0.75**	
BEP	0.00	0.00	0.00	0.00	0.00	0.00	0.00
ε*_wax-xylem-alkane_	Lycop	Fern	Gymn	Magn	Eudic	Monoc(-)	Poal (-)
Fern	**0.29**						
Gymn	0.01	**0.47**					
Magn	0.00	0.03	**0.77**				
Eudic	0.00	**0.20**	**1.00**	**0.46**			
Monoc(-)	0.00	0.00	0.03	**1.00**	0.00		
Poal(-)	0.00	0.00	0.00	**0.64**	0.00	**0.68**	
BEP	0.00	0.00	0.00	0.00	0.00	0.00	0.01

Lycop  =  Lycopod; Gymn  =  Gymnosperm; Magn  =  Magnoliids; Eudic  =  Eudicots; Monoc(-)  =  Monocots excluding Poales; and Poal(-). =  Poales excluding BHP. The values in bold show the corresponding two group means are not significantly different at 95% confidence level. The ANOVA test suggests that Magnoliids are not significantly different from eudicots, gymnosperms, monocots (-) and Poales(-) (in the case of *n*-alkanes), although this could be a result of small sample numbers (3) for this plant group.

When explicitly mapped on a phylogeny, our dataset shows strong phylogenetic patterning that more closely related species also have more similar fractionation values (Fig. 2; Fig. S5; Table 1; Table S5). The ‘Lambda metric’ analyses recovered high levels of phylogenetic signal that were statistically significant as assessed by permutation tests (Lambda  = 0.83, p<0.001). These results confirm the visual pattern observed on the tree. All monocots exhibit very large fractionation, and within monocots, the largest is clustered still, in the BEP clade of grasses (Fig. 1; Fig. 2; Fig. S5). These visual patterns were confirmed by modeling the evolution of hydrogen isotopic fractionation under three distinctive optima: one for BEP grasses, one for all non-BEP monocots, and one for all non-monocot vascular plants. This model was a significantly better fit than a single or two optimum model or a Brownian motion model of trait evolution [26], [27] (Table 1; “Methods” Section). Overall, centering and scaling method provide better presentation of data for leaf lipid δD analysis. It provides integrated data to represent individual plants, which can be readily incorporated to phylogenetic examination. It also presents better separation between lineages. In general, uncertainty within lineages are reduced (e.g., lycopods, Poales, etc), which helps the detection of distinct difference between lineages.

### Leaf wax hydrogen isotope fractionation in plants with different growth forms

**Figure 3:**
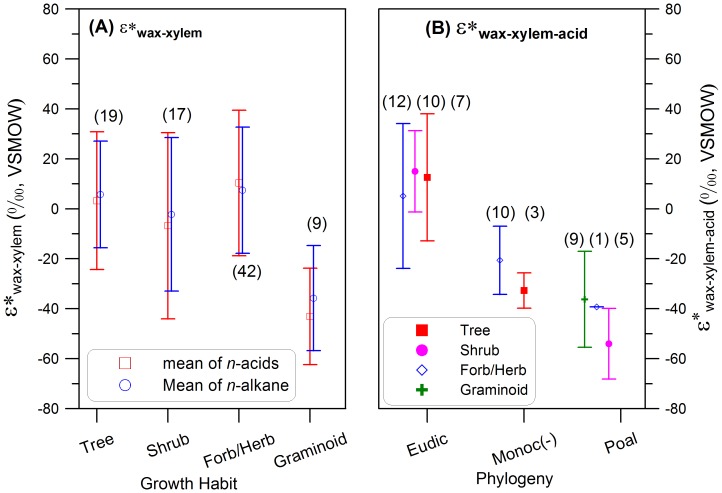
Comparison of *ε_wax-xylem_ values (scaled mean values): A) plants are classified by growth forms as shrubs, trees, forb/herb and graminoid; and B) plant are classified by their phylogenetic lineages including Poales, monocots (excluding Poales) and eudicots. The error bars show the 1 σ standard deviation for all the species in individual lineages. Numbers on the charts denote the species numbers sampled for individual lineages or types.

Conversely, if we plot our data from NYBG in terms of plant growth forms (Fig. 3A), shrub, trees, and forbs do not show statistically different fractionation. In cases where plants exhibit different growth forms but belong to the same major phylogenetic clade, fractionation values are similar (Fig. 3B), indicating plant growth forms are not the principal determinant for the fractionation values. For example, palm trees (monocot trees) have similar fractionation values to those of forb/herbs in Liliaceae (monocot herbs). Tree ferns have significantly smaller fractionation than palm trees, but have similar values to those of other ferns (Fig. 2; Fig. S5) [10]. The highest fractionation values within the ‘graminoids’ are found in bamboos, which might be better described as having a shrub-like habit, with multiple, tall growing shoots.

When including graminoids as a plant growth form, a standard ANOVA analysis supports an effect of growth form on the absolute ε*_wax-water_ values (F = 6.91, p<0.01). However, ‘graminoids’ is a standard growth form category used by ecologists that actually refers to a plant lineage, in this case grasses and sedges. Thus, the perceived hydrogen isotopic difference between different plant growth forms is primarily due to the coincidence of graminoids as a growth form that occurs solely in the monocots, which is a phylogenetic clade with distinct fractionation.

### Influence of photosynthetic pathways

**Figure 4:**
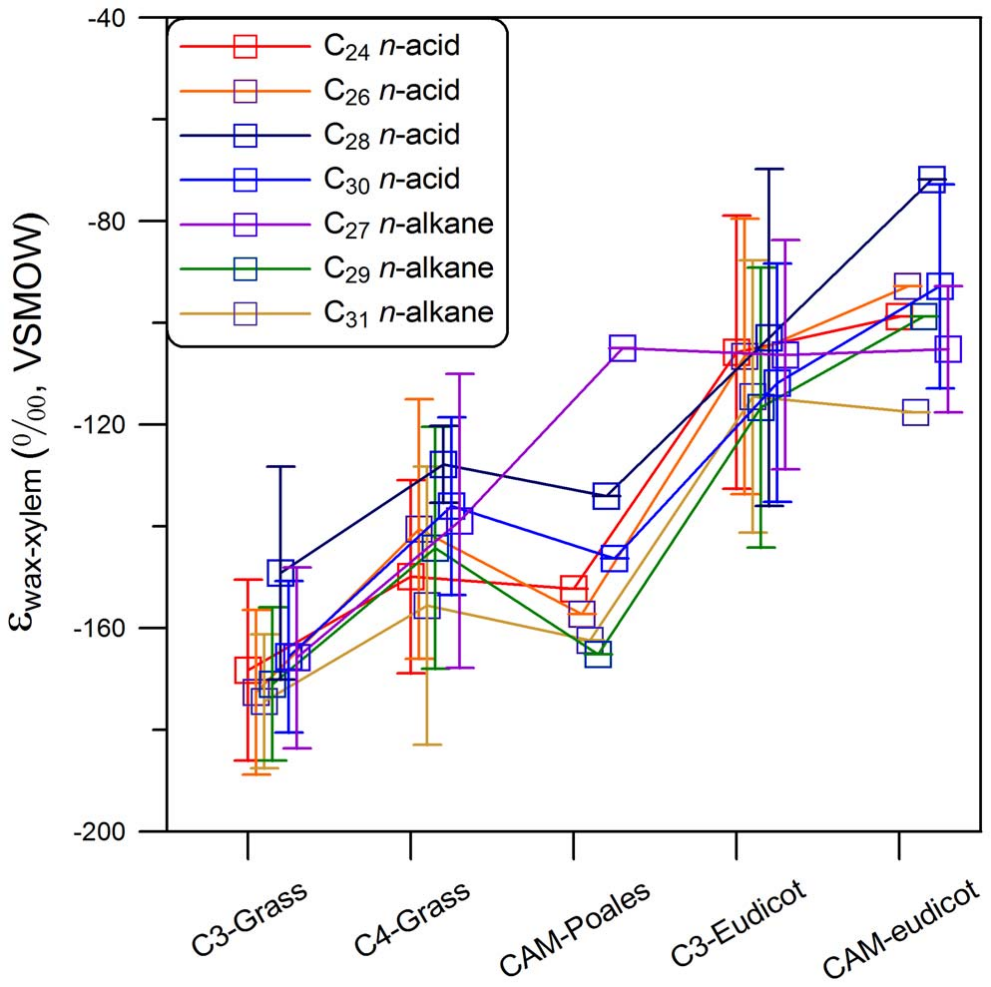
Comparison of leaf wax hydrogen isotope fractionation values relative to xylem water in different phylogenetic lineages with different photosynthetic pathways. The error bars show the 1 σ standard deviation for all the species in individual lineages.

In contrast to the strong phylogenetic patterns of leaf wax hydrogen isotopic fractionation, photosynthetic pathways exert a minor impact, as found in many previous studies [28]–. Leaf wax hydrogen isotope fractionation relative to xylem water (absolute values) in C_4_ grasses is ∼20 ‰ (17 ‰ in the composite dataset from [13]) smaller than that in C_3_ grasses (Fig. 4). Such difference is thought to originate from different venation patterns and physiological characteristics between C_3_ and C_4_ grasses [28]. Similarly, species exhibiting Crassulacean Acid Metabolism (CAM) show smaller absolute leaf wax hydrogen isotope fractionation values than C_3_ species within individual major lineages (Fig. 4), though our sample number of CAM species is admittedly small (a total of 5 species). Despite the hydrogen isotopic fractionation difference between C_3_ and C_4_ plants, C_4_ grasses still have ∼30 ‰ larger fractionation than C_3_ eudicots. Therefore, isotope effects derived from photosynthetic methods are subordinate to the very large difference between monocots and other plants. Fractionation in C_4_ grasses (n = 3) was ∼30 ‰ smaller than that in C_3_ grasses (n = 11) (Fig 4), although this could also be largely a clade effect, as 10 of our 11 C_3_ grasses were sampled from the BEP lineage (Fig. 1; Fig. 2; Fig. S5).

## Discussions

### Possible factors that contribute to the phylogenetic patterns in plant leaf wax hydrogen isotopic fractionation

**Figure 5:**
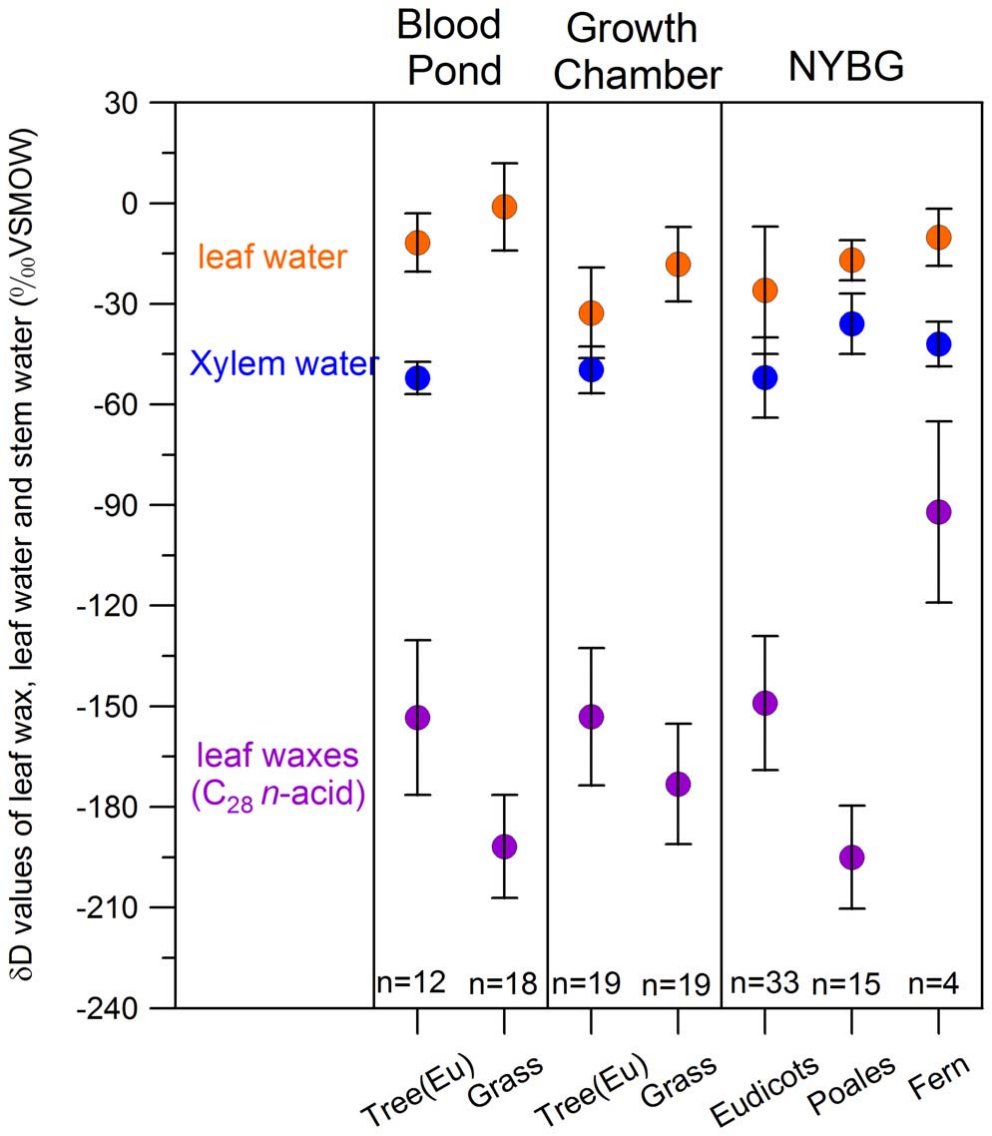
Comparison in δD values of leaf water, xylem water and leaf waxes for plant samples collected in natural environments (Blood Pond, Massachusetts, and NYBG), respectively) and grown in growth chambers. Only C_28_ *n*-acid is shown for comparison (other *n*-acid compounds show similar patterns). The error bars show the 1 σ standard deviation for all the plant samples in individual dataset. Numbers show the species numbers for individual dataset. Note in all cases (120 samples excluding ferns), grasses have higher leaf waters δD values, but lower leaf wax δD values, than trees. The data suggest, in addition to leaf water, differences in biosynthetic fractionation between grasses and eudicot trees are also important in controlling the observed difference in hydrogen isotopic fractionation.

What are the mechanisms underlying these strong phylogenetic patterns? From xylem water to leaf waxes, there are numerous biosynthetic steps that may impart different hydrogen isotopic fractionation [13], [31], and unraveling the exact mechanism will require comprehensive examination of each point where reactants are not 100% transformed into products. One explanation would be that plants have different evapotranspiration rates, as has been suggested previously [10], [13], [32], and recently further demonstrated by analyzing plant leaf water from across a large relative humidity gradient [33]–[34]. To test if leaf water δD difference can fully explain the contrasting leaf wax δD difference between eudicots and grasses we analyzed a total of 110 eudicot and grass samples from growth chamber or field (Table S2; Fig.5; Fig. S7). We find in all cases, grasses have ∼10 to 15 ‰ higher leaf water δD values than eudicots, but ∼30 to 50 ‰ lower leaf wax δD values than eudicots (Fig. 5). Additionally, the leaf water δD values in our plant samples from NYBG vary only ∼16 ‰ and show little correlation with leaf wax δD values (Table S1). For example, four fern species have slightly higher leaf water δD values than Poales, but have much heavier leaf wax δD values than Poales (Fig.5). We do acknowledge, however, that sampling leaf water may carry inherent uncertainties because of the variable nature of leaf isotopic composition during the diurnal cycle [35], though we did sample our leaves at nearly the same time of day (12pm–2pm). While leaf water δD values must affect the leaf wax isotopic values [16], [18], our results suggest other factors are likely also important in determining the different hydrogen isotopic fractionation between eudicot trees and grasses (Fig. 5, Fig. S7).

Another explanation for the isotopic difference among different lineages is that plants have evolved divergent strategies of allocating photosynthetic products among metabolic processes. Our recent studies of leaf wax regeneration rates from a monocot (the grass *Phleum pratense*) and a eudicot (the tree *Fraxinus americana*) in greenhouse experiments show that leaf wax production appears to begin significantly earlier in the grass during a diurnal cycle [16], [18]. If eudicots in general tend to show lower rates of wax regeneration, their isotopic signal may be controlled primarily by the rapid leaf expansion phase in the early growth season [16], [18], [32], [36]–[37]. Such scenario is consistent with our data of naturally occurring plants collected around Blood Pond in May, July and September, respectively (Fig. S7). Leaf waxes from eudicot trees remained relatively constant over the growth season, probably mostly inheriting an early season isotopic signal [18], and grasses maintain lower δD values than trees throughout the season (Table S2, Fig. S7). These observations suggest that synthesis of leaf waxes in eudicots likely use isotopically enriched sugars that are already partially used for other metabolic and biosynthetic functions, or stored in roots and trunks over the winter season, contributing to the relatively high leaf wax δD values [38]. We also concur with the proposal of Kahmen et al. [33] that differential degree of hydrogen atoms derived from water versus from NADPH may also be important in regulating the hydrogen isotopic fractionation of plant leaf waxes.

### Evolution and leaf wax hydrogen isotope fractionation

If our experiments with grasses and trees can be extrapolated more broadly, it is possible that the observed phylogenetic trend is related to a change in the rate and timing of leaf wax synthesis during seasonal and/or diurnal cycles. Such changes in biosynthetic strategy may have resulted in differences in prioritizing leaf wax synthesis and utilization of fresh/residual sugars. Additionally, leaf wax synthesis could have been significantly altered by the evolution of the monocot leaf, which is developmentally very different than leaves in other plant groups, with the leaf lamina possibly being derived from the leaf base [39] and also having an extended period of growth and development via an intercalary meristem. Our observations of exceedingly high isotopic fractionation in the ‘BEP’ grass lineage have no reasonable explanation at this time, and warrant more research.

In summary, our study shows clearly that growth form or plant ‘functional type’ bears little on values of hydrogen fractionation in leaf waxes. Variation in hydrogen isotopic fractionation among vascular plants is best explained by plant lineage differences, and may reflect, at least in part, evolutionarily driven changes in biosynthetic priorities and leaf developmental programs. Paleoclimate reconstruction utilizing leaf wax δD values must take into consideration these significant isotopic differences between plant lineages.

## Supporting Information

Figure S1 **Sampling species numbers in each major phylogenetic lineage (data from Sachse et al (2012) for the upper panel and this study for the lower panel).** The published sampling pool, reviewed by Sachse et al (2012), is heavily biased toward Poales and Eudicots.
(DOC)

Figure S2 **Comparison of chain-length distributions of n-alkanes between all trees of any phylogenetic lineages and all Poaceae and between C_4_ Poaceae and C_3_ Poaceae from NYBG.** n-Alkanes of majority of trees and grasses are dominated by C_29_ n-alkanes, whereas 4 out of 9 C_3_ grasses and 2 out of 3 C_4_ grasses have C_31_ n-alkane as the most abundant n-alkane lipid. The error bars show the 1 σ standard deviation for each leaf lipid.
(DOC)

Figure S3 **Correlation of hydrogen isotope fractionation values between individual leaf lipids and xylem water.**
(DOC)

Figure S4 **The Principle Component Analysis (using MatLab) of the δD data of 4 individual n-acids and 3 individual n-alkanes.**
(DOC)

Figure S5 **Leaf wax hydrogen isotope fractionation values for all plant species from NYBG on a phylogenetic tree.** The tip values are the combined scaled values of n-alkanes ε*wax-xylem-alkane, based on measurements of collected modern species (as in Fig. 1), and branches are colored according to the inferred ancestral values of ε*_wax-xylem-alkane_ across the phylogenetic tree.
(DOC)

Figure S6 **Replotting three datasets by phylogeny (This study; Sachse et al (2012); and Hou et al (2007), respectively).** Only hydrogen isotope fractionations of C_29_ n-alkane and C_28_ n-acid relative to mean annual precipitation (MAP) are shown for comparison, while other leaf wax compounds show similar patterns. δD values of MAP were calculated from the Online Precipitation Isotopes Calculator. The error bars show the 1 σ standard deviation for all the available species in individual lineages. Numbers show the species numbers for individual lineages. The gray boxes represent the box-whisker plots, whereas the magenta lines represent category mean values with 1 σ standard deviation.
(DOC)

Figure S7 **Seasonal variations in δD values of leaf water, xylem water and leaf waxes for plant samples collected from Blood Pond, Massachusetts (USA).** Only C_28_ n-acid is shown for comparison, while other n-acids show similar patterns. Note in all cases, grasses have higher leaf water δD values, but lower leaf wax δD values than trees (Table S2). The error bars show the 1 σ standard deviation for all the species in each season.
(DOC)

Table S1 δD values of leaf waxes, leaf water and xylem water for all the plant samples from the NYBG. C_24_, C_26_, C_28_ and C_30_ are n-alkanoic acids and C_27_, C_29_ and C_30_ are n-alkanes. Leaf δD and xylem δD values are for water distilled from leaf and stem sampled during 12pm-2pm of the sampling day, respectively. Lycopods (lycop), Gymnosperm (gymn), Magnoliids (magn), Eudicots (eudic), monocots (monoc), Poales (Poal); Forb/herb (F/H), climbing vine (CV) and Graminoids (Gram).
(DOC)

Table S2 The δD values of leaf lipid C_28_ n-acid, leaf water and xylem water for the field (Blood Pond) and growth chamber plant samples. The left side are for tree species and the right are for grasses. The irrigation water used for growth chamber experiments has a δD value of −49 ‰.
(DOC)

Table S3 The leaf lipid abundances for each n-alkane lipid for plant samples collected from the New York Botanic Garden. The unit for single lipids and sums is µg/g d.w. leaf. ACL is the average chain length (ACL =  ∑n*Cn/sum, where n is carbon number 21, 23,25,27,29, 31 and 33, Cn is lipid with n carbon number and sum is total mass of C21–C33 n-alkanes). R is the ratio of total n-acids (C20–C32) to total n-alkanes (C21–C33).
(DOC)

Table S4 The leaf lipid abundances for each n-alkanoic acid lipid for plant samples collected from the New York Botanic Garden. The unit for single lipids and sums is µg/g d.w. leaf. ACL is the average chain length (ACL =  ∑n*Cn/sum, where n is carbon number 24, 26, 28, 30, and 32, Cn is lipid with n carbon number and sum is total mass of C24–C32 n-acids).
(DOC)

Table S5 The centered and VAST-scaled ε*_wax-xylem_ values for the 7 leaf lipids for each plant species. The detailed description for the scaling and combination is described in “Methods”.
(DOC)

## References

[1] Pagani M, Pedentchouk N, Huber M, Sluijs A, Schouten S, et al (2006) Arctic hydrology during global warming at the Palaeocene/Eocene thermal maximum. Nature 442(7103): 671–675. 16906647 10.1038/nature05043

[2] Huang Y, Clemens SC, Liu W, Wang Y, Prell WL (2007) Large-scale hydrological change drove the late Miocene C_4_ plant expansion in the Himalayan foreland and Arabian Peninsula. Geology 35(6): 531–534.

[3] Tierney JE, Russell JM, Huang Y, Sinninghe Damsté JS, Hopmans EC, Cohen AS (2008) Northern Hemisphere Controls on Tropical Southeast African Climate During the Past 60,000 Years. Science 322(5899): 252–255. 18787132 10.1126/science.1160485

[4] Polissar PJ, Freeman KH, Rowley DB, McInerney FA, Currie BS (2009) Paleoaltimetry of the Tibetan Plateau from D/H ratios of lipid biomarkers. Earth Planet Sci Lett 287(1–2): 64–76.

[5] Schefuß E, Kuhlmann H, Mollenhauer G, Prange M, Patzold J (2011) Forcing of wet phases in southeast Africa over the past 17,000 years. Nature 480(7378): 509–512. 22193106 10.1038/nature10685

[6] Dupont LM, Rommerskirchen F, Mollenhauer G, Schefuß E (2013) Miocene to Pliocene changes in South African hydrology and vegetation in relation to the expansion of C_4_ plants,. Earth Plan Sci Let 375: 408–417 10.1016/j.epsl.2013.06.005.

[7] Kuechler RR, Schefuß E, Beckmann B, Dupont L, Wefer G (2013) NW African hydrology and vegetation during the Last Glacial cycle reflected in plant-wax-specific hydrogen and carbon isotopes,. Quat. Sci. Rev. 82: 56–67 ISSN 0277-3791, 10.1016/j.quascirev.2013.10.013.

[8] Rach O, Brauer A, Wilkes H, Sachse D (2014) Delayed hydrological response to Greenland cooling at the onset of the Younger Dryas in western Europe,. Nature Geoscience 7: 109–112 10.1038/ngeo2053

[9] Zhuang G, Brandon MT, Pagani M, Krishnan S (2014) Leaf wax stable isotopes from Northern Tibetan Plateau: Implications for uplift and climate since 15 Ma,. Earth Planet Sci Lett 390: 186–198.

[10] Hou J, D'Andrea WJ, MacDonald D, Huang Y (2007) Hydrogen isotopic variability in leaf waxes among terrestrial and aquatic plants around Blood Pond, Massachusetts (USA). Org Geochem 38(6): 977–984.

[11] Liu W, Yang H (2008) Multiple controls for the variability of hydrogen isotopic compositions in higher plant n-alkanes from modern ecosystems. Global Change Biol 14(9): 2166–2177.

[12] Gao L, Hou J, Toney J, MacDonald D, Huang Y (2011) Mathematical modeling of the aquatic macrophyte inputs of mid-chain n-alkyl lipids to lake sediments: Implications for interpreting compound specific hydrogen isotopic records. Geochim Cosmochim Acta 75(13): 3781–3791.

[13] Sachse D, Billault I, Bowen G, Chikaraishi Y, Dawson T, et al (2012) Molecular Paleohydrology: Interpreting the Hydrogen-Isotopic Composition of Lipid Biomarkers from Photosynthesizing Organisms. Annu Rev Earth Planet Sci 40(1): 221–249.

[14] Edwards EJ, Still CJ, Donoghue MJ (2007) The relevance of phylogeny to studies of global change. Trends Ecol Evol 22(5): 243–249. 17296242 10.1016/j.tree.2007.02.002

[15] Bowen GJ, Revenaugh J (2003) Interpolating the isotopic composition of modern meteoric precipitation. Water Resour Res 39(10).

[16] Gao L, Burnier A, Huang Y (2012a) Quantifying instantaneous regeneration rates of plant leaf waxes using stable hydrogen isotope labeling. Rapid Comm Mass Spectrom 26(2): 115–122. 10.1002/rcm.5313 22173799

[17] Hou J, D'Andrea WJ, Huang Y (2008) Can sedimentary leaf waxes record D/H ratios of continental precipitation? Field, model, and experimental assessments. Geochim Cosmochim Acta 72: 3503–3517 10.1016/j.gca.2008.04.030

[18] Gao L, Tsai YJ, Huang Y (2012b) Assessing the rate and timing of leaf wax regeneration in Fraxinus americana using stable hydrogen isotope labeling. Rapid Comm. Mass Spectrom 26(19): 2241–50 10.1002/rcm.6348 22956315

[19] Gao L, Zheng M, Fraser M, Huang Y (2014) Comparable hydrogen isotopic fractionation of plant leaf wax n-alkanoic acids in arid and humid subtropical ecosystems,. Geochem Geophys Geosyst 15: 361–373.

[20] Keun HC, Ebbels TMD, Antti H, Bollard ME, Beckonert O, Holmes E, Lindon JC, Nicholson JK (2003) Improved analysis of multivariate data by variable stability scaling: application to NMR-based metabolic profiling. Analytica Chimica Acta 490(1–2): 265–276.

[21] Eriksson L, Johansson E, Kettaneh-Wold H, Wold S (1999) Introduction to Multi and Megavariate Analysis using Projection Methods (PCA and PLS), UMETRICS Inc., Umeå, 1999.

[22] Hellberg S, Eriksson L, Jonsson J, Lindgren F, Sjöström M, Skagerberg B, Wold S, Andrews P (1991) Minimum analogue peptide sets (MAPS) for quantitative structure-activity relationships. Int J Pept Prot Res 37 (1991): 414–424. 10.1111/j.1399-3011.1991.tb00756.x 1917297

[23] Webb CO, Ackerly DD, Kembel SW (2008) Phylocom: software for the analysis of phylogenetic community structure and trait evolution. Bioinformatics 24: 2098–2100. 18678590 10.1093/bioinformatics/btn358

[24] Pagel M (1999) Inferring the historical patterns of biological evolution. Nature 401: 877–884. 10553904 10.1038/44766

[25] Revell LJ (2012) Phytools: An R package for phylogenetic comparative biology (and other things). Methods Ecol Evol 3: 217–223 10.1111/j.2041-210X.2011.00169.x

[26] Paradis E, Claude J, Strimmer K (2004) APE: analyses of phylogenetics and evolution in R language. Bioinformatics 20: 289–290. 14734327 10.1093/bioinformatics/btg412

[27] Butler MA, King AA (2004) Phylogenetic comparative analysis: a modeling approach for adaptive evolution. Amer Nat 164: 683–695. 29641928 10.1086/426002

[28] Smith FA, Freeman KH (2006) Influence of physiology and climate on δD of leaf wax n-alkanes from C_3_ and C_4_ grasses. Geochim Cosmochim Acta 70(5): 1172–1187.

[29] Feakins SJ, Sessions AL (2010) Crassulacean acid metabolism influences D/H ratio of leaf wax in succulent plants. Org Geochem 41(12): 1269–1276.

[30] McInerney FA, Helliker BR, Freeman KH (2011) Hydrogen isotope ratios of leaf wax n-alkanes in grasses are insensitive to transpiration. Geochim Cosmochim Acta 75(2): 541–554.

[31] Shepherd T, Griffiths DW (2006) The effects of stress on plant cuticular waxes. New Phytologist 171(3): 469–499. 16866954 10.1111/j.1469-8137.2006.01826.x

[32] Gao L, Huang Y (2012) Inverse gradients in leaf wax δD and δ^13^C values along grass blades of Miscanthus sinensis: Implications for leaf wax reproduction and plant physiology. Oecologia 10.1007/s00442-012-2506-6 23132687

[33] Kahmen A, Schefuß E, Sachse D (2013a) Leaf water deuterium enrichment shapes leaf wax n-alkane δD values of angiosperm plants I: Experimental evidence and mechanistic insights,. Geochim Cosmochim Acta 111: 39–49 ISSN 0016-7037, 10.1016/j.gca.2012.09.003.

[34] Kahmen A, Hoffmann B, Schefuß E, Arndt SK, Cernusak CA, West JB, Sachse D (2013b) Leaf water deuterium enrichment shapes leaf wax n-alkane δD values of angiosperm plants II: Observational evidence and global implications,. Geochim Cosmochim Acta 111: 50–63 ISSN 0016-7037, 10.1016/j.gca.2012.09.004.

[35] Cernusak LA, Pate JS, Farquhar GD (2002) Diurnal variation in the stable isotope composition of water and dry matter in fruiting *Lupinus angustifolius* under field conditions. Plant, Cell Environ 25 (7): 893–907.

[36] Kahmen A, Dawson TE, Vieth A, Sachse D (2011) Leaf wax *n*-alkane δD values are determined early in the ontogeny of Populus trichocarpa leaves when grown under controlled environmental conditions. Plant, Cell Environ 34(10): 1639–1651. 21696403 10.1111/j.1365-3040.2011.02360.x

[37] Tipple BJ, Berke MA, Doman CE, Khachaturyan S, Ehleringer JR (2013) Leaf-wax *n*-alkanes record the plant–water environment at leaf flush. Earth Planet Sci Lett 110 (7): 2659–2664 10.1073/pnas.1213875110 PMC3574905 23359675

[38] Shchepinov MS (2007) Do “heavy” eaters live longer? BioEssays 29(12): 1247–1256. 18027392 10.1002/bies.20681

[39] Kaplan DR (1973) The problem of leaf morphology and evolution in the mono- cotyledons. Quarterly Rev Biol 48: 437–457.

