# Supplementary material for: Major Evolutionary Trends in Hydrogen Isotope Fractionation of Vascular Plant Leaf Waxes
Source: PLoS One. 2014 Nov 17;9(11):e112610. doi: 10.1371/journal.pone.0112610 (PMC4234459; doi:10.1371/journal.pone.0112610)
Supplement: Figure S1 — Sampling species numbers in each major phylogenetic lineage (data from Sachse et al (2012) for the upper panel and this study for the lower panel). The published sampling pool, reviewed by Sachse et al (2012), is heavily biased toward Poales and Eudicots. (DOC) [file pone.0112610.s001.doc]

**Figure S1.** Sampling species numbers in each major phylogenetic lineage (data from Sachse et al (2012) for the upper panel and this study for the lower panel). The published sampling pool, reviewed by Sachse et al (2012), is heavily biased toward Poales and Eudicots.


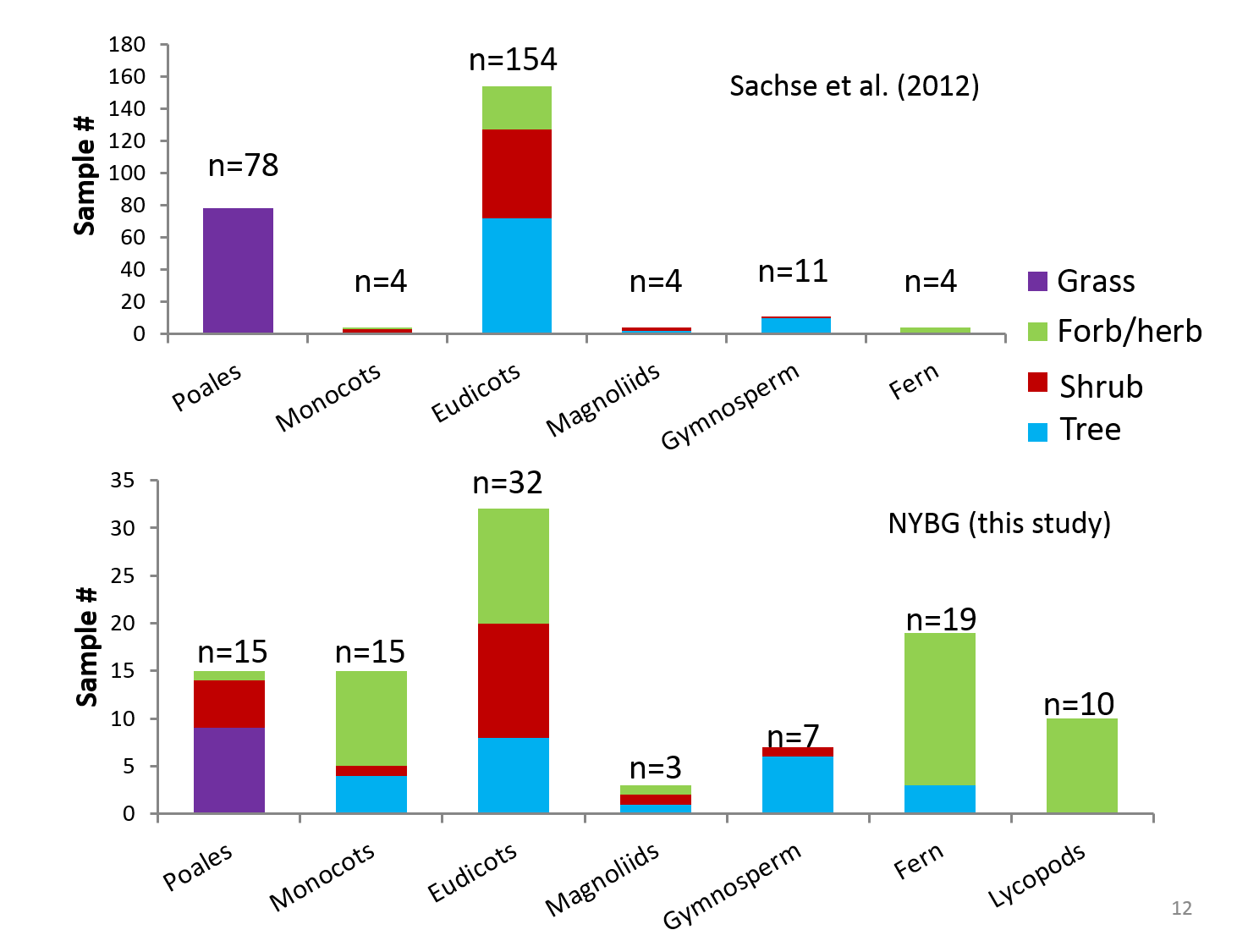
­­­­

**REFERENCES**

Sachse D., Billault I., Bowen G. J., Chikaraishi Y., Dawson T. E., Feakins S. J., Freeman K. H., Magill C. R., McInerney F. A., van der Meer M. T. J., Polissar P., Robins R. J., Sachs J. P., Schmidt H.-L., Sessions A. L., White J. W. C., West J. B. and Kahmen A. (2012) Molecular paleohydrology: Interpreting the hydrogen-isotopic composition of lipid biomarkers from photosynthesizing organisms. *Annual Review of Earth and Planetary Sciences* **40**, 221-249.
