# Supplementary material for: Major Evolutionary Trends in Hydrogen Isotope Fractionation of Vascular Plant Leaf Waxes
Source: PLoS One. 2014 Nov 17;9(11):e112610. doi: 10.1371/journal.pone.0112610 (PMC4234459; doi:10.1371/journal.pone.0112610)

**Figure S2.** Comparison of chain-length distributions of *n*-alkanes between all trees of any phylogenetic lineages and all Poaceae and between C_4_ Poaceae and C_3_ Poaceae from NYBG. *n*-Alkanes of majority of trees and grasses are dominated by C_29_ *n*-alkanes, whereas 4 out of 9 C_3_ grasses and 2 out of 3 C_4_ grasses have C_31_ *n*-alkane as the most abundant *n*-alkane lipid. The error bars show the 1 σ standard deviation for each leaf lipid.


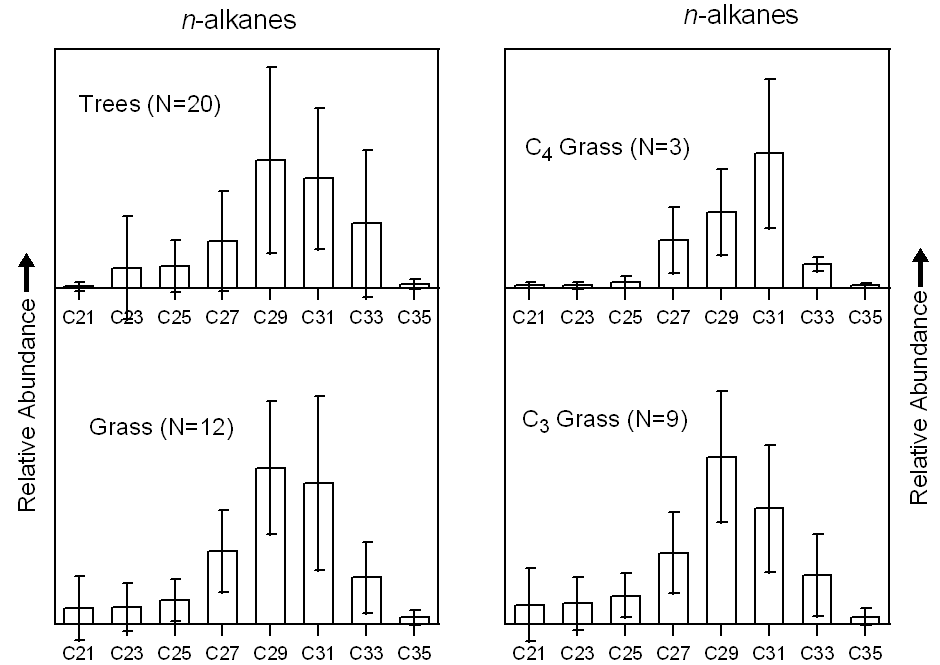

Supplement: Figure S2 — Comparison of chain-length distributions of n-alkanes between all trees of any phylogenetic lineages and all Poaceae and between C4 Poaceae and C3 Poaceae from NYBG. n-Alkanes of majority of trees and grasses are dominated by C29 n-alkanes, whereas 4 out of 9 C3 grasses and 2 out of 3 C4 grasses have C31 n-alkane as the most abundant n-alkane lipid. The error bars show the 1 σ standard deviation for each leaf lipid. (DOC) [file pone.0112610.s002.doc]
