# Supplementary material for: Major Evolutionary Trends in Hydrogen Isotope Fractionation of Vascular Plant Leaf Waxes
Source: PLoS One. 2014 Nov 17;9(11):e112610. doi: 10.1371/journal.pone.0112610 (PMC4234459; doi:10.1371/journal.pone.0112610)

**Figure S3.** Correlation of hydrogen isotope fractionation values between individual leaf lipids and xylem water.


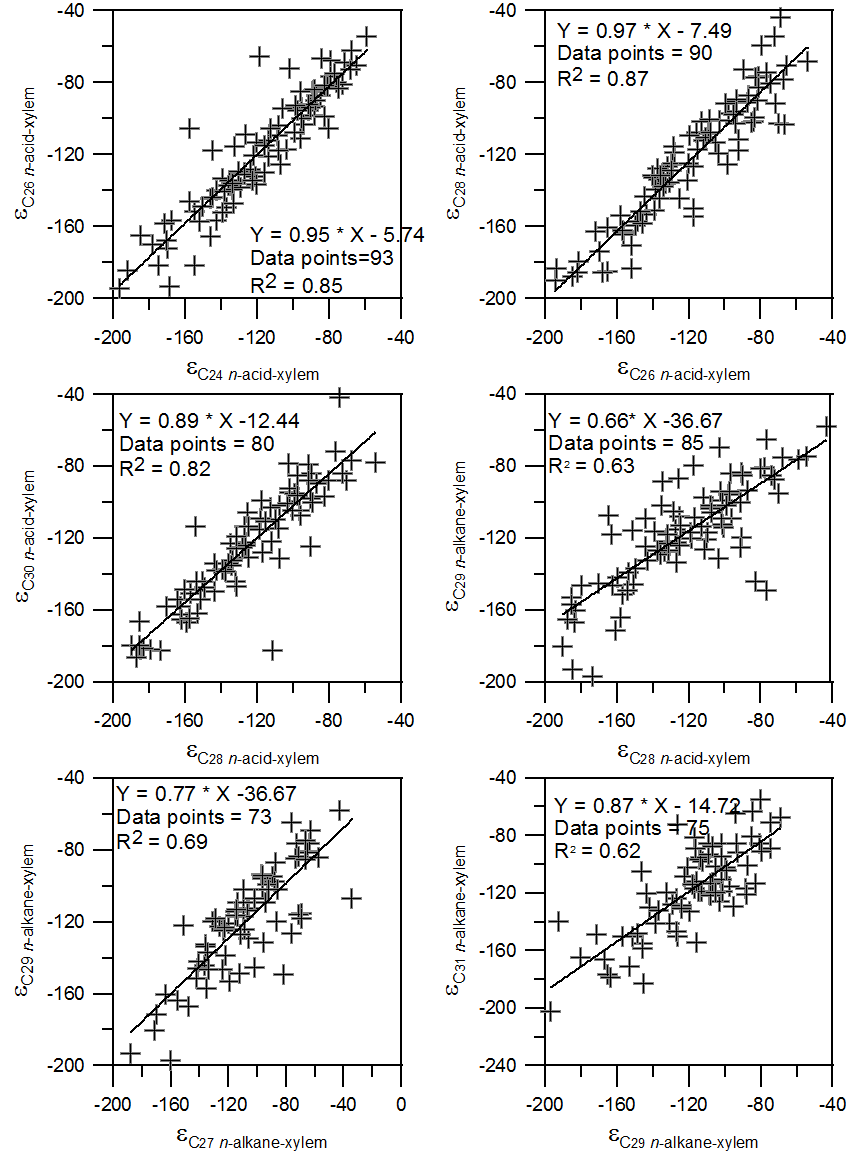

Supplement: Figure S3 — Correlation of hydrogen isotope fractionation values between individual leaf lipids and xylem water. (DOC) [file pone.0112610.s003.doc]
