# Supplementary material for: Major Evolutionary Trends in Hydrogen Isotope Fractionation of Vascular Plant Leaf Waxes
Source: PLoS One. 2014 Nov 17;9(11):e112610. doi: 10.1371/journal.pone.0112610 (PMC4234459; doi:10.1371/journal.pone.0112610)

**Figure S4.** The Principle Component Analysis (using MatLab) of the δD data of 4 individual *n*-acids and 3 individual *n*-alkanes.


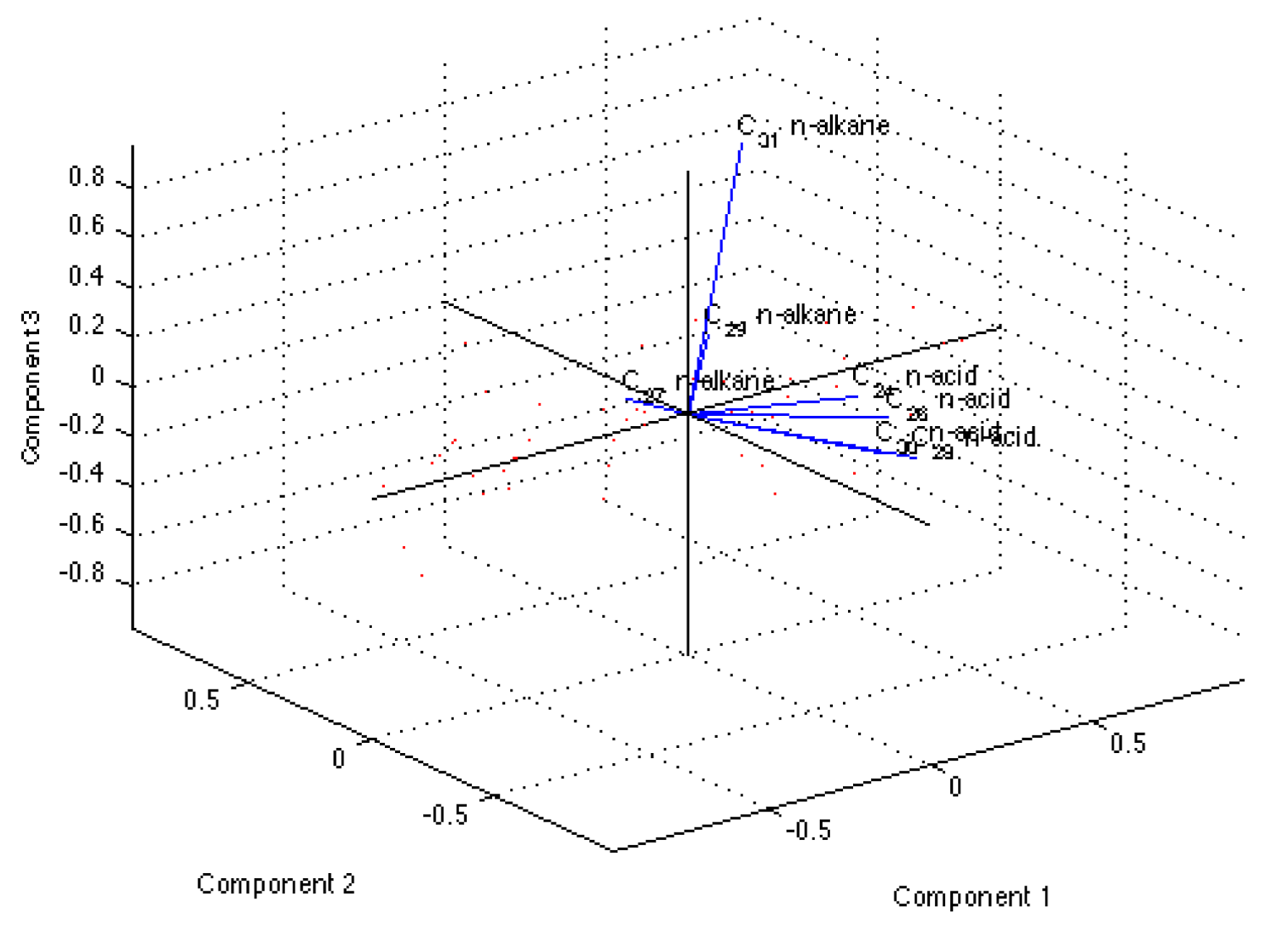

Supplement: Figure S4 — The Principle Component Analysis (using MatLab) of the δD data of 4 individual n-acids and 3 individual n-alkanes. (DOC) [file pone.0112610.s004.doc]
