# Supplementary material for: Major Evolutionary Trends in Hydrogen Isotope Fractionation of Vascular Plant Leaf Waxes
Source: PLoS One. 2014 Nov 17;9(11):e112610. doi: 10.1371/journal.pone.0112610 (PMC4234459; doi:10.1371/journal.pone.0112610)
Supplement: Figure S5 — Leaf wax hydrogen isotope fractionation values for all plant species from NYBG on a phylogenetic tree. The tip values are the combined scaled values of n-alkanes ε*wax-xylem-alkane, based on measurements of collected modern species (as in Fig. 1), and branches are colored according to the inferred ancestral values of ε*wax-xylem-alkane across the phylogenetic tree. (DOC) [file pone.0112610.s005.doc]

**Figure S5.** Leaf wax hydrogen isotope fractionation values for all plant species from NYBG on a phylogenetic tree. The tip values are the combined scaled values of n-alkanes ε*wax-xylem-alkane, based on measurements of collected modern species (as in Fig. 1), and branches are colored according to the inferred ancestral values of ε*_wax-xylem-alkane_ across the phylogenetic tree.

**
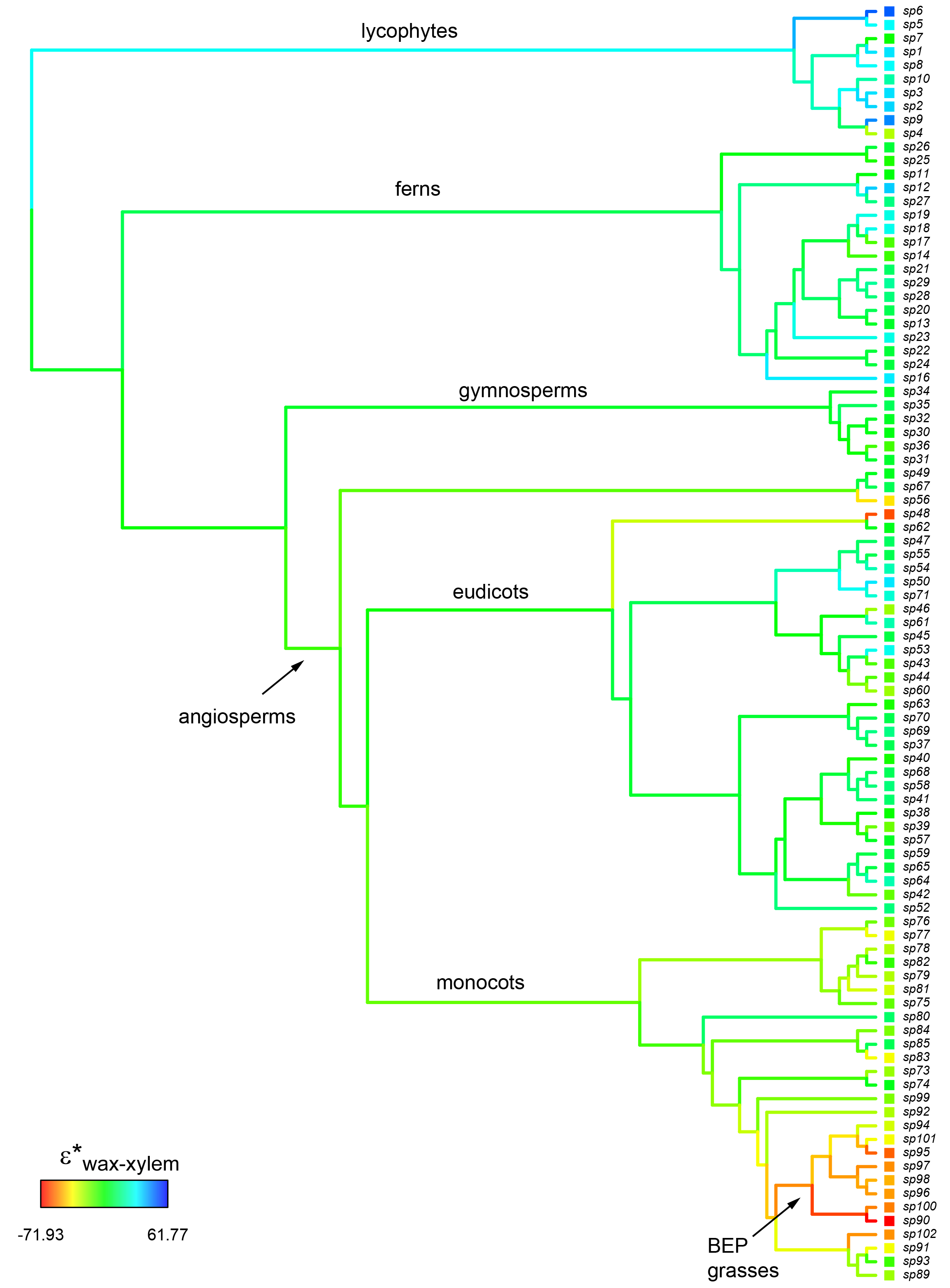
**
