# Supplementary material for: Major Evolutionary Trends in Hydrogen Isotope Fractionation of Vascular Plant Leaf Waxes
Source: PLoS One. 2014 Nov 17;9(11):e112610. doi: 10.1371/journal.pone.0112610 (PMC4234459; doi:10.1371/journal.pone.0112610)
Supplement: Figure S6 — Replotting three datasets by phylogeny (This study; Sachse et al (2012); and Hou et al (2007), respectively). Only hydrogen isotope fractionations of C29 n-alkane and C28 n-acid relative to mean annual precipitation (MAP) are shown for comparison, while other leaf wax compounds show similar patterns. δD values of MAP were calculated from the Online Precipitation Isotopes Calculator. The error bars show the 1 σ standard deviation for all the available species in individual lineages. Numbers show the species numbers for individual lineages. The gray boxes represent the box-whisker plots, whereas the magenta lines represent category mean values with 1 σ standard deviation. (DOC) [file pone.0112610.s006.doc]

**Figure S6.** Replotting three datasets by phylogeny (This study; Sachse et al (2012); and Hou et al (2007), respectively). Only hydrogen isotope fractionations of C_29_ *n*-alkane and C_28_ *n*-acid relative to mean annual precipitation (MAP) are shown for comparison, while other leaf wax compounds show similar patterns. δD values of MAP were calculated from the Online Precipitation Isotopes Calculator. The error bars show the 1 σ standard deviation for all the available species in individual lineages. Numbers show the species numbers for individual lineages. The gray boxes represent the box-whisker plots, whereas the magenta lines represent category mean values with 1 σ standard deviation.


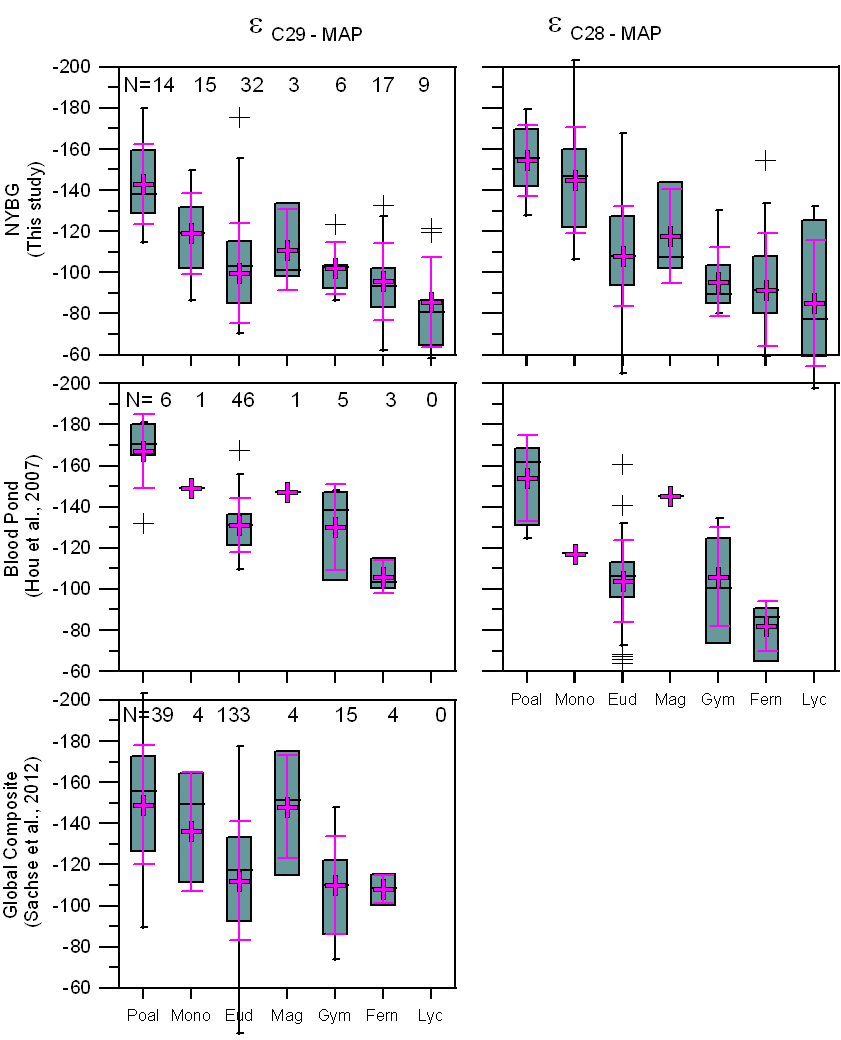


**REFERENCES**

Sachse D., Billault I., Bowen G. J., Chikaraishi Y., Dawson T. E., Feakins S. J., Freeman K. H., Magill C. R., McInerney F. A., van der Meer M. T. J., Polissar P., Robins R. J., Sachs J. P., Schmidt H.-L., Sessions A. L., White J. W. C., West J. B. and Kahmen A. (2012) Molecular paleohydrology: Interpreting the hydrogen-isotopic composition of lipid biomarkers from photosynthesizing organisms. *Annual Review of Earth and Planetary Sciences* **40**, 221-249.

Hou, J., D'Andrea, W.J., MacDonald, D., Huang, Y. (2007) Hydrogen isotopic variability in leaf waxes among terrestrial and aquatic plants around Blood Pond, Massachusetts (USA). *Org. Geochem.* **38**(6):977-984.
