# Supplementary material for: Major Evolutionary Trends in Hydrogen Isotope Fractionation of Vascular Plant Leaf Waxes
Source: PLoS One. 2014 Nov 17;9(11):e112610. doi: 10.1371/journal.pone.0112610 (PMC4234459; doi:10.1371/journal.pone.0112610)

**Figure S7.** Seasonal variations in δD values of leaf water, xylem water and leaf waxes for plant samples collected from Blood Pond, Massachusetts (USA). Only C_28_ *n*-acid is shown for comparison, while other *n*-acids show similar patterns. Note in all cases, grasses have higher leaf water δD values, but lower leaf wax δD values than trees (Table S2-SIA). The error bars show the 1 σ standard deviation for all the species in each season.


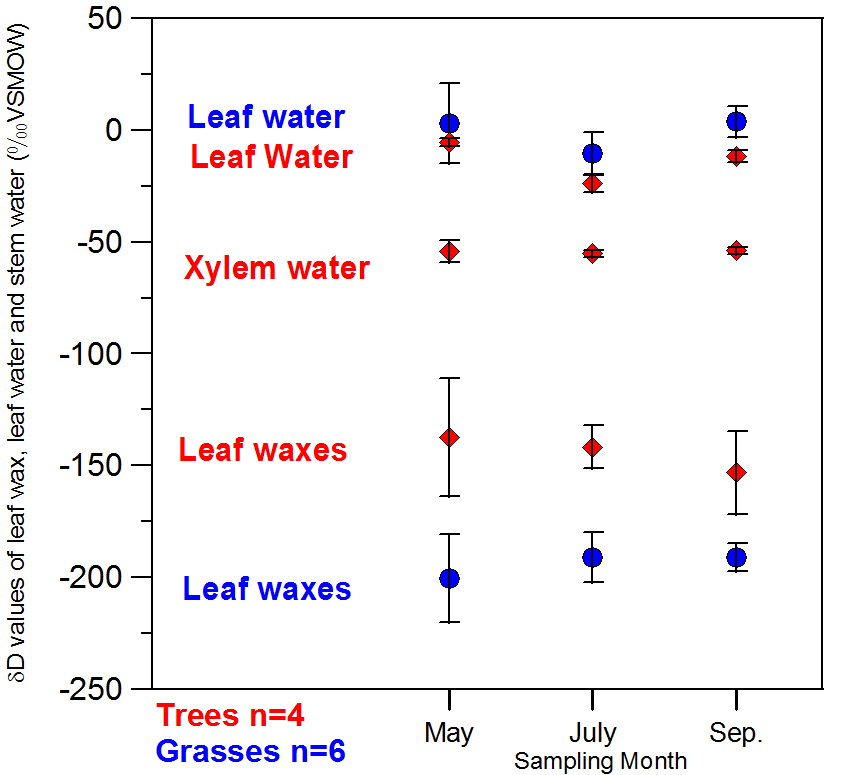

Supplement: Figure S7 — Seasonal variations in δD values of leaf water, xylem water and leaf waxes for plant samples collected from Blood Pond, Massachusetts (USA). Only C28 n-acid is shown for comparison, while other n-acids show similar patterns. Note in all cases, grasses have higher leaf water δD values, but lower leaf wax δD values than trees (Table S2). The error bars show the 1 σ standard deviation for all the species in each season. (DOC) [file pone.0112610.s007.doc]
