# Supplementary material for: Major Evolutionary Trends in Hydrogen Isotope Fractionation of Vascular Plant Leaf Waxes
Source: PLoS One. 2014 Nov 17;9(11):e112610. doi: 10.1371/journal.pone.0112610 (PMC4234459; doi:10.1371/journal.pone.0112610)
Supplement: Table S1 — δD values of leaf waxes, leaf water and xylem water for all the plant samples from the NYBG. C24, C26, C28 and C30 are n-alkanoic acids and C27, C29 and C30 are n-alkanes. Leaf δD and xylem δD values are for water distilled from leaf and stem sampled during 12pm-2pm of the sampling day, respectively. Lycopods (lycop), Gymnosperm (gymn), Magnoliids (magn), Eudicots (eudic), monocots (monoc), Poales (Poal); Forb/herb (F/H), climbing vine (CV) and Graminoids (Gram). (DOC) [file pone.0112610.s008.doc]

**Table S1.**  δD values of leaf waxes, leaf water and xylem water for all the plant samples from the NYBG. C_24_, C_26_, C_28_ and C_30_ are *n*-alkanoic acids and C_27_, C_29_ and C_30_ are *n*-alkanes. Leaf δD and xylem δD values are for water distilled from leaf and stem sampled during 12pm-2pm of the sampling day, respectively. Lycopods (lycop), Gymnosperm (gymn), Magnoliids (magn), Eudicots (eudic), monocots (monoc), Poales (Poal); Forb/herb (F/H), climbing vine (CV) and Graminoids (Gram).

| **ID** | **Lineages** | **Scientific Name** | **Growth**  **Form** | **C24** | **C26** | **C28** | **C30** | **C27** | **C29** | **C31** | **Leaf-δD** | **Xylem-δD** |
| --- | --- | --- | --- | --- | --- | --- | --- | --- | --- | --- | --- | --- |
| sp1 | Lycop | *Selaginella moellendorffii* | F/H | -141 | -137 |  |  | -117 | -129 |  |  |  |
| sp2 | Lycop | *Selaginella huehuetenangensis* | F/H | -132 | -121 | -131 |  | -119 | -133 | -109 |  |  |
| sp3 | Lycop | *Selaginella schiedeana* | F/H | -125 | -134 | -129 | -124 | -129 | -118 |  |  |  |
| sp4 | Lycop | *Selaginella kraussiana* | F/H | -192 | -189 | -182 | -193 | -200 | -172 |  |  |  |
| sp5 | Lycop | *Huperzia squarrosa* | F/H | -129 | -132 | -113 |  | -126 | -129 |  |  |  |
| sp6 | Lycop | *Huperzia sp.* | F/H | -124 | -122 | -98 |  | -98 | -112 |  |  |  |
| sp7 | Lycop | *Selaginella mosorongensis* | F/H | -137 | -139 | -143 | -175 | -139 | -170 | -182 |  |  |
| sp8 | Lycop | *Selaginella mickelii* | F/H | -132 | -129 | -127 | -96 | -126 | -137 | -117 |  |  |
| sp9 | Lycop | *Selaginella galeottii* | F/H |  |  |  |  | -101 |  |  |  |  |
| sp10 | Lycop | *Selaginella brauniii* | F/H | -177 | -179 | -175 |  | -139 | -139 |  |  |  |
| sp11 | Fern | *Dicksonia antartica* | Tree | -171 | -155 | -147 | -141 |  | -143 | -164 | -17.6 | -44.0 |
| sp12 | Fern | *Alsophila firma* | Tree | -111 | -117 | -148 | -124 | -110 | -116 | -114 | -1.9 | -49.9 |
| sp13 | Fern | *Matteuccia struthiopteris* | F/H | -123 | -127 | -129 | -117 | -128 | -127 | -160 | -3.7 | -34.7 |
| sp14 | Fern | *Adiantum pedatum* | F/H | -111 | -119 | -113 |  | -118 | -182 |  | -17.5 | -38.8 |
| sp15 | Fern* | *Marattia attenuata* | F/H | -155 | -168 | -203 | -164 |  |  |  |  |  |
| sp16 | Fern* | *Dennstaedtia globulifera* | F/H | -140 | -150 | -148 | -156 | -111 | -136 |  |  |  |
| sp17 | Fern* | *Asplenium formosanum* | F/H | -184 | -186 | -183 | -182 |  | -177 | -174 |  |  |
| sp18 | Fern* | *Thelypteris* sp*.* | F/H | -130 | -127 | -133 | -140 | -118 | -133 | -137 |  |  |
| sp19 | Fern* | *Blechnum glandulosum* | F/H | -147 | -145 | -142 | -149 | -124 | -136 |  |  |  |
| sp20 | Fern* | *Arachniodes amabilis* | F/H | -164 | -163 | -163 | -172 | -140 | -149 | -165 |  |  |
| sp21 | Fern* | *Arthropteris articulata* | F/H | -153 | -125 | -143 | -131 |  | -151 | -153 |  |  |
| sp22 | Fern* | *Bolbitis portoricensis* | F/H | -140 | -145 | -141 | -151 |  |  | -157 |  |  |
| sp23 | Fern* | *Campyloneurum angustifolium* | F/H | -133 | -134 | -142 | -136 | -120 | -137 | -133 |  |  |
| sp24 | Fern* | *Tectaria zeylanica* | F/H | -160 | -160 | -159 | -165 | -148 | -153 | -161 |  |  |
| sp25 | Fern* | *Osmunda regalis* | F/H | -148 | -162 | -161 | -160 | -171 | -171 | -154 |  |  |
| sp26 | Fern* | *Osmundastrum cinnamomeum* | F/H | -142 | -145 | -148 | -158 | -165 | -160 | -137 |  |  |
| sp27 | Fern* | *Sphaeropteris horrida* | Tree | -169 | -119 | -123 | -140 | -141 | -146 |  |  |  |
| sp28 | Fern* | *Davallia fejeensis* 'Plumosa' | F/H | -139 | -139 | -139 | -141 | -149 | -145 | -143 |  |  |
| sp29 | Fern* | *Davallia trichomanoides* | F/H | -140 | -142 | -125 | -136 |  | -139 | -152 |  |  |
| sp30 | Gymn | *Taxodium distichum* | Tree | -135 | -144 | -141 |  | -155 | -154 | -137 | -9.5 | -44.8 |
| sp31 | Gymn | *Abies homolepis* | Tree |  | -147 | -137 |  | -141 | -144 | -109 | -9.1 | -30.9 |
| sp32 | Gymn | *Metasequoia glyptostroboides* | Tree | -162 | -155 | -143 |  | -148 | -154 | -140 | -15.7 | -46.8 |
| sp33 | Gymn | *Picea orientalis* | Tree | -158 | -141 | -133 | -137 |  |  |  | -9.2 | -36.0 |
| sp34 | Gymn | *Ginkgo biloba* | Tree | -197 | -147 | -154 | -145 | -149 | -155 | -138 | -11.4 | -46.7 |
| sp35 | Gymn | *Ephedra gerardiana* | Shrub | -129 | -124 | -141 |  | -133 | -139 | -143 | -9.7 | -43.7 |
| sp36 | Gymn | *Pinus parviflora* | Tree | -183 | -177 | -180 | -178 | -151 | -173 | -172 | -13.5 | -50.7 |
| sp37 | Eudic | *Bougainvillea* 'Tahitian Dawn' | CV | -149 | -138 | -154 | -147 | -147 | -161 | -146 | -87.9 | -58.3 |
| sp38 | Eudic | *Amsonia tabernaemontana* | F/H | -152 | -169 | -187 | -178 | -154 | -154 | -165 | -53.0 | -50.1 |
| sp39 | Eudic | *Galium odoratum* | F/H | -152 | -156 | -147 | -138 | -160 | -148 |  | -21.1 | -32.3 |
| sp40 | Eudic | *Salvia transsylvanica* | F/H | -149 | -145 | -160 | -156 | -151 | -157 | -157 | -30.2 | -46.2 |
| sp41 | Eudic | *Nicotiana mutabilis* | F/H | -166 | -170 | -158 | -153 |  |  | -141 | *-41.3* | -47.5 |
| sp42 | Eudic | *Rudbeckia maxima* | F/H | -160 | -171 | -166 | -162 | -165 | -162 |  | -41.3 | -44.6 |
| sp43 | Eudic | *Euphorbia collorata* | F/H | -128 | -111 | -146 | -143 | -138 | -172 | -182 | -16.1 | -47.4 |
| sp44 | Eudic | *Baptisia australis* | F/H | -138 | -142 | -135 | -131 |  | -166 |  | -39.8 | -47.4 |
| sp45 | Eudic | *Sanguisorba obtusa* | F/H | -149 | -145 | -160 | -156 | -147 | -162 | -178 | -22.5 | -66.9 |
| sp46 | Eudic | *Geranium* 'Brookside' | F/H | -173 | -172 | -170 | -184 |  | -165 | -187 | -9.3 | -44.0 |
| sp47 | Eudic | *Paeonia* 'Lovebirds' | F/H | -161 | -167 | -180 | -181 |  | -137 | -164 | -17.8 | -52.8 |
| sp48 | Eudic | *Dicentra spectabilis* | F/H | -215 | -196 | -215 | -212 | -218 | -222 | -171 | -60.5 | -36.7 |
| sp50 | Eudic | *Rodgersia podophylla* | F/H | -118 | -122 | -105 | -127 | -116 | -124 | -120 | -15.8 | -53.3 |
| sp51 | Eudic | *Genipa americana* | Tree | -150 | -116 | -121 | -121 |  | -123 |  | -70.4 |  |
| sp52 | Eudic | *Nyssa sylvatica* | Tree | -136 | -137 | -155 | -223 | -122 | -169 | -118 | -16.5 | -49.4 |
| sp53 | Eudic | *Hevea brasiliensis* | Tree | -123 | -118 | -131 | -139 |  | -137 | -150 | -18.9 | -67.2 |
| sp54 | Eudic | *Cercidiphyllum japonicum* | Tree | -156 | -149 |  |  | -81 | -150 | -155 | -13.5 | -48.2 |
| sp55 | Eudic | *Liquidambar styraciflua* | Tree | -135 | -143 | -143 | -165 |  | -141 | -137 | -14.1 | -39.4 |
| sp57 | Eudic | *Coffea arabica* | Shrub | -119 | -134 | -160 | -141 |  | -143 | -148 | -9.9 | -39.3 |
| sp58 | Eudic | *Brunfelsia pilosa* | Shrub | -104 | -98 |  | -139 | -128 | -136 | -121 | -38.7 | -38.6 |
| sp59 | Eudic | *Ilex americana* | Tree | -176 | -177 | -168 | -154 | -112 | -156 | -159 | -29.0 | -46.1 |
| sp60 | Eudic | *Prosopis glandulosa* | Tree | -147 | -146 | -147 | -160 | -197 | -204 | -182 | -23.8 | -70.0 |
| sp61 | Eudic | *Cotinus coggygria '*Royal Purple' | Shrub | -149 | -138 | -146 | -136 |  | -137 | -130 | -16.5 | -48.1 |
| sp62 | Eudic | *Bocconia frutescens* | Tree | -168 | -176 | -177 | -175 | -154 | -155 | -129 | -19.4 | -44.0 |
| sp63 | Eudic | *Simmondsia chinensis* | Shrub | -183 | -181 | -177 | -167 | -168 | -166 | -172 | -10.4 | -60.0 |
| sp64 | Eudic | *Angelica gigas* | Shrub | -144 | -142 | -144 | -142 | -143 | -140 | -112 | -29.3 | -51.3 |
| sp65 | Eudic | *Oreopanax capitatus* | Shrub | -189 | -193 | -197 | -185 | -173 | -165 | -158 | -14.6 | -70.9 |
| sp66 | Eudic | *Macleania insignis* | Shrub | -140 | -156 | -179 | -166 | -117 | -141 | -153 | -8.4 |  |
| sp68 | Eudic | *Brunfelsia pauciflora* | Shrub | -121 | -145 |  | -188 |  | -129 | -160 | -30.4 | -44.2 |
| sp69 | Eudic | *Oscularia deltoides* | Shrub |  |  |  |  |  | -126 | -155 | 2.7 | -47.4 |
| sp70 | Eudic | *Alluaudia humbertii* | Shrub |  |  |  |  | -111 | -154 | -150 | -3.6 | -42.7 |
| sp71 | Eudic | *Crassula ovata* | Shrub | -137 | -131 | -155 | -137 |  | -119 | -129 | 2.3 | -42.4 |
| sp72 | Eudic | *Crassula muscosa* | Shrub | -157 | -147 | -163 | -164 | -69 | -111 | -138 |  |  |
| sp49 | Magn | *Laurus nobilis* | Tree | -196 | -194 | -193 | -188 | -186 | -183 | -171 | -18.1 | -58.2 |
| sp56 | Magn | *Asarum europaeum* | F/H | -150 | -148 | -153 | -158 | -138 | -152 | -156 | -13.5 | -32.0 |
| sp67 | Magn | *Piper betle* | Shrub | -157 | -156 | -158 | -169 |  | -150 | -162 | -12.8 | -46.5 |
| sp73 | Monoc | *Dichorisandra thyrsiflora* | F/H | -187 | -186 | -195 | -203 | -166 | -181 | -184 | -8.6 | -49.5 |
| sp74 | Monoc | *Costus barbatus* | F/H | -155 | -168 | -167 | -164 |  | -145 | -158 | 1.8 | -44.2 |
| sp75 | Monoc | *Iris* sp*.* | F/H | -176 | -191 | -199 | -204 | -179 | -180 | -186 | *-37.1* | -63.6 |
| sp76 | Monoc | *Spathoglottis plicata* | F/H | -165 | -176 | -179 | -180 | -154 | -169 | -188 | -0.9 | -48.3 |
| sp77 | Monoc | *Vanda tricolor var. planilabris* | F/H | -140 | -146 | -157 | -151 | -148 | -152 | -161 | 9.0 | -7.6 |
| sp78 | Monoc | *Hosta plantaginea* 'Aphrodite' | F/H | -193 | -205 | -214 | -221 | -197 | -197 | -186 | -32.6 | *-64.7* |
| sp79 | Monoc | *Convallaria majalis* | F/H | -148 | -157 | -166 | -163 | -175 | -172 |  | -16.8 | -44.8 |
| sp80 | Monoc | *Lilium* 'Pink Twinkle' *asiatic hybrid lilly* | F/H | -188 | -202 | -207 | -203 | -119 | -165 |  | -33.8 | -53.3 |
| sp81 | Monoc | *Allium christophii* | F/H | -203 | -200 | -218 | -206 | -154 | -194 | -230 | -19.6 | -57.2 |
| sp82 | Monoc | *Polygonatum odoratum var. pluriflorum* 'Variegatum' | F/H | -184 | -186 | -178 | -172 | -170 | -169 | -156 | -46.2 | -53.1 |
| sp83 | Monoc | *Chamaedorea pochutlensis* | Tree | -197 | -196 | -204 | -197 | -190 | -198 | -197 | -13.8 | -55.2 |
| sp84 | Monoc | *Rhapis humilis* | Tree | -175 | -188 | -193 | -195 |  | -159 | -195 | -12.8 | -48.5 |
| sp85 | Monoc | *Attalea oleveira* | Tree | -211 | -200 | -208 | -198 | -148 | -153 | -134 | -19.0 | -51.5 |
| sp86 | Monoc | *Mauritiella armata* | Tree | -164 | -160 | -172 | -137 |  | -139 | -185 | -13.4 |  |
| sp87 | Monoc | *Sabal etonia* | Shrub | -191 | -196 | -249 | -183 | -162 | -163 |  | -8.3 |  |
| sp88 | BEP | *Phalaris arundinacea var. picta* 'Picta' | Gram | -196 | -198 | -189 | -189 |  |  |  | -9.3 | -31.3 |
| sp89 | Poal | *Miscanthus sinensis* 'Gracillimus' | Gram | -177 | -150 | -181 | -179 | -167 | -169 | -164 | -13.5 | *-37.2* |
| sp90 | BEP | *Stipa calamagrostis* | Gram | -209 | -201 | -205 | -213 | -192 | -227 | -232 | -11.5 | *-37.2* |
| sp91 | Poal | *Hakonechloa macra* 'Aureola' | Gram | -196 | -193 | -210 | -191 | -146 | -178 | -195 | -27.2 | -29.8 |
| sp92 | Poal | *Carex oshimensis* | Gram | -208 | -195 | -191 | -182 | -152 | -186 | -185 | *-14.8* | -43.8 |
| sp93 | Poal | *Cymbopogon citratus* | Gram | -180 | -182 | -178 | -166 | -175 | -165 | -161 | -23.4 | -53.1 |
| sp94 | BEP | *Lithachne pauciflora* | Gram | -198 | -204 | -202 | -205 | -149 | -170 | -182 | -15.9 | -27.7 |
| sp95 | BEP | *Chusquea liebmannii* | Shrub | -223 | -221 | -217 | -207 | -199 | -207 | -192 | -24.9 | -33.2 |
| sp96 | BEP | *Phyllostachys nigra* | Shrub | -187 | -205 | -201 | -189 | -210 | -211 | -189 | -19.4 | -47.5 |
| sp97 | BEP | *Indocalamus tessellatus* | Shrub | -184 | -197 | -226 | -224 | -208 | -204 |  | -13.7 | -52.5 |
| sp98 | BEP | *Phyllostachys aureosulcata* | Shrub | -209 | -233 | -223 |  | -190 | -208 | -207 | -21.4 | -48.9 |
| sp99 | Poal | *Alcantarea imperialis* | F/H | -194 | -198 | -203 | -206 | -176 | -188 | -149 | -24.5 | -48.9 |
| sp100 | BEP | *Leymus arenarius* | Gram | -227 | -220 | -223 | -221 |  | -201 | -212 | -17.3 | -43.3 |
| sp101 | BEP | *Chusquea virgata* | Shrub | -182 | -209 | -212 | -206 | -163 | -185 | -178 | -14.4 | -32.7 |
| sp102 | Poal | *Zeugites americana* | Gram | -178 | -167 | -179 | -185 | -176 | -184 | -198 | -19.0 | -24.4 |
