# Supplementary material for: Major Evolutionary Trends in Hydrogen Isotope Fractionation of Vascular Plant Leaf Waxes
Source: PLoS One. 2014 Nov 17;9(11):e112610. doi: 10.1371/journal.pone.0112610 (PMC4234459; doi:10.1371/journal.pone.0112610)
Supplement: Table S2 — The δD values of leaf lipid C28 n-acid, leaf water and xylem water for the field (Blood Pond) and growth chamber plant samples. The left side are for tree species and the right are for grasses. The irrigation water used for growth chamber experiments has a δD value of −49 ‰. (DOC) [file pone.0112610.s009.doc]

**Table S2.** The δD values of leaf lipid C_28_ *n*-acid, leaf water and xylem water for the field (Blood Pond) and growth chamber plant samples. The left side are for tree species and the right are for grasses. The irrigation water used for growth chamber experiments has a δD value of -49 ‰.

| **species** | **C_28_** | **Leaf water** | **Xylem water** | **Sampling Date** |  | **species** | **C_28_** | **Leaf water** | **Sampling Date** |
| --- | --- | --- | --- | --- | --- | --- | --- | --- | --- |
| **Hemlock^a^** | -168 | -6 | -50 | 05/22/07 |  | **Orchard grass** | -165 | 16 | 05/22/07 |
| **White pine** | -188 | -2 | -56 | 05/22/07 |  | **Sweet vernal grass** | -194 | 9 | 05/22/07 |
| **Red maple** | -105 | -3 | -54 | 05/22/07 |  | **Velvet grass** | -221 | 20 | 05/22/07 |
| **Red oak** | -167 | -7 | -48 | 05/22/07 |  | **Rye grass** | -204 | -29 | 06/07/07 |
| **Black birch** | -147 | -6 | -59 | 05/22/07 |  | **Crabgrass** | -206 | 7 | 06/21/07 |
| **White ash** | -130 | -6 | -55 | 05/22/07 |  | **Timothy** | -212 | -5 | 06/28/07 |
| **Hemlock^b^** | -177 | -17 | -45 | 06/30/07 |  | **Orchard grass** | -193 | -20 | 06/30/07 |
| **White pine** | -193 | -20 | -48 | 06/30/07 |  | **Sweet vernal grass** | -201 | -17 | 06/30/07 |
| **Red maple** | -152 | -26 | -57 | 06/30/07 |  | **Velvet grass** | |  | 06/30/07 |
| **Red oak** | -135 | -27 | -54 | 06/30/07 |  | **Rye grass** | -191 |  | 06/30/07 |
| **Black birch** | -147 | -24 | -57 | 06/30/07 |  | **Crabgrass** | -172 | 0 | 06/30/07 |
| **White ash** | -132 | -19 | -54 | 06/30/07 |  | **Timothy** | -198 | -5 | 06/30/07 |
| **Hemlock^c^** | -169 | 1 | -42 | 09/21/07 |  | **Orchard grass** | -194 | 1 | 09/21/07 |
| **White pine** | -137 | -3 | -45 | 09/21/07 |  | **Sweet vernal grass** | -197 | 0 | 09/21/07 |
| **Red maple** | -149 | -11 | -56 | 09/21/07 |  | **Velvet grass** | |  | 09/21/07 |
| **Red oak** | -140 | -13 | -52 | 09/21/07 |  | **Rye grass** | |  | 09/21/07 |
| **Black birch** | -144 | -15 | -53 | 09/21/07 |  | **Crabgrass** | -184 | 14 | 09/21/07 |
| **White ash** | -180 | -9 | -54 | 09/21/07 |  | **Timothy** | -188 | 1 | 09/21/07 |
|  |  |  |  |  |  |  |  |  |  |
| **Alberta spruce^d^** | -155 | -38 | -69 |  |  | **Foxtail** | -167 | -40 |  |
| **Alberta spruce** | -138 | -15 | -18 |  |  | **Foxtail** | -173 | -6 |  |
| **Alberta spruce** | -141 | -26 | -52 |  |  | **Foxtail** | -167 | -16 |  |
| **White cedar** | -159 | -43 | -82 |  |  | **Foxtail** | -164 | -28 |  |
| **White cedar** | -136 | -26 | -48 |  |  | **Foxtail** | -163 | -22 |  |
| **White cedar** | -140 | -25 | -50 |  |  | **Foxtail** | -161 | -9 |  |
| **Red maple** | -157 | -55 | -48 |  |  | **Orchard grass** | -189 | -43 |  |
| **Red maple** | -143 | -36 | -46 |  |  | **Orchard grass** | -192 | -16 |  |
| **Red maple** | -136 | -28 | -47 |  |  | **Orchard grass** | -194 | -16 |  |
| **Red oak** | -177 | -35 |  |  |  | **Orchard grass** | -194 | -11 |  |
| **Red oak** | -184 | -17 | -42 |  |  | **Orchard grass** | -203 | -9 |  |
| **Red oak** | -159 | -32 |  |  |  | **Orchard grass** | -211 | -13 |  |
| **Red oak** | -183 | -18 |  |  |  | **Timothy weed** | -173 | -10 |  |
| **Red oak** | -172 | -32 |  |  |  | **Timothy weed** | -162 | -7 |  |
| **White ash** | -122 | -7 | -41 |  |  | **Timothy weed** | -159 | -21 |  |
| **White ash** | -141 | -45 | -60 |  |  | **Timothy weed** | -158 | -34 |  |
| **White ash** | -138 | -52 |  |  |  | **Timothy weed** | -152 | -25 |  |
| **White ash** | -145 | -32 | -57 |  |  | **Timothy weed** | -152 | -9 |  |
| **White ash** | -134 | -37 | -56 |  |  | **Timothy weed** | -158 | -12 |  |

^a,b,c^ Data in black, red and green colors are for the field samples collected in May, July and September, respectively.

^d^ Data in blue are for the growth chamber data under constant environment conditions (see detailed procedures in the “Materials and Methods”).
