# Supplementary material for: Major Evolutionary Trends in Hydrogen Isotope Fractionation of Vascular Plant Leaf Waxes
Source: PLoS One. 2014 Nov 17;9(11):e112610. doi: 10.1371/journal.pone.0112610 (PMC4234459; doi:10.1371/journal.pone.0112610)
Supplement: Table S3 — The leaf lipid abundances for each n-alkane lipid for plant samples collected from the New York Botanic Garden. The unit for single lipids and sums is µg/g d.w. leaf. ACL is the average chain length (ACL = ∑n*Cn/sum, where n is carbon number 21, 23,25,27,29, 31 and 33, Cn is lipid with n carbon number and sum is total mass of C21–C33 n-alkanes). R is the ratio of total n-acids (C20–C32) to total n-alkanes (C21–C33). (DOC) [file pone.0112610.s010.doc]

**Table S3.** The leaf lipid abundances for each *n*-alkane lipid for plant samples collected from the New York Botanic Garden. The unit for single lipids and sums is µg/g d.w. leaf. ACL is the average chain length (ACL= ∑n*Cn /sum, where n is carbon number 21, 23,25,27,29, 31 and 33, Cn is lipid with n carbon number and sum is total mass of C21-C33 n-alkanes). R is the ratio of total n-acids (C20-C32) to total n-alkanes (C21-C33).

| **ID** |  | **C21** | **C23** | **C25** | **C27** | **C29** | **C31** | **C33** | **R** | **Sum**  **C21-33** | **ACL C21-33** |
| --- | --- | --- | --- | --- | --- | --- | --- | --- | --- | --- | --- |
| 1 | Hosta plantaginea 'Aphrodite' | 4.2 | 2.5 | 9.6 | 8.6 | 7.1 | 2.7 | 0.2 | 6.5 | 34.8 | 26.2 |
| 2 | Polygonatum odoratum var. pluriflorum 'Variegatum' | 0.4 | 10.4 | 52.0 | 62.5 | 190.1 | 5.3 | 0.2 | 1.3 | 320.9 | 27.8 |
| 4 | Miscanthus sinensis 'Gracillimus' | 1.7 | 0.7 | 3.6 | 19.0 | 35.6 | 23.3 | 6.1 | 2.8 | 89.9 | 29.0 |
| 5 | Stipa calamagrostis | 0.3 | 2.3 | 6.1 | 8.7 | 30.7 | 35.0 | 1.3 | 3.4 | 84.6 | 29.2 |
| 6 | Allium christophii | 22.2 | 13.1 | 31.0 | 92.4 | 176.5 | 2871.5 | 0.0 | 0.1 | 3206.6 | 30.6 |
| 7 | Rodgersia podophylla | 0.4 | 7.3 | 10.9 | 19.5 | 111.9 | 67.4 | 1.7 | 0.8 | 219.0 | 29.1 |
| 8 | Dicentra spectabilis | 0.8 | 1.1 | 9.0 | 181.2 | 333.2 | 10.8 | 3.3 | 3.4 | 539.4 | 28.3 |
| 9 | Convallaria majalis | 0.4 | 5.2 | 23.9 | 22.1 | 13.4 | 0.8 | 0.1 | 3.5 | 65.9 | 26.4 |
| 10 | Lilium 'Pink Twinkle' asiatic hybrid lilly | 0.1 | 9.7 | 43.0 | 80.3 | 606.2 | 17.0 | 1.1 | 0.4 | 757.5 | 28.5 |
| 11 | Matteuccia struthiopteris | 0.2 | 2.6 | 28.0 | 20.4 | 9.8 | 10.6 | 2.8 | 1.3 | 74.4 | 27.2 |
| 12 | Hakonechloa macra 'Aureola' | 5.6 | 9.2 | 10.3 | 15.5 | 44.7 | 314.4 | 30.9 | 0.3 | 430.5 | 30.3 |
| 13 | Asarum europaeum | 0.0 | 0.4 | 1.2 | 0.7 | 0.7 | 1.1 | 0.2 | 16.0 | 4.3 | 27.7 |
| 14 | Adiantum pedatum | 0.0 | 0.4 | 1.3 | 4.0 | 4.1 | 7.6 | 52.2 | 4.0 | 69.5 | 32.0 |
| 15 | Carex oshimensis | 0.7 | 8.1 | 7.9 | 3.7 | 25.3 | 7.1 | 4.4 | 4.5 | 57.2 | 27.9 |
| 16 | Nicotiana mutabilis | 0.4 | 1.0 | 13.5 | 44.7 | 48.2 | 315.7 | 839.2 | 0.0 | 1262.6 | 32.0 |
| 17 | Iris sp. | 0.5 | 2.2 | 7.2 | 7.8 | 29.4 | 30.0 | 18.1 | 0.1 | 95.1 | 29.8 |
| 18 | Baptisia australis | 0.0 | 3.3 | 13.3 | 22.1 | 288.4 | 5.0 | 0.4 | 0.5 | 332.5 | 28.7 |
| 19 | Salvia transsylvanica | 0.0 | 0.5 | 3.2 | 41.5 | 124.0 | 256.7 | 161.5 | 0.2 | 587.5 | 30.8 |
| 20 | Euphorbia collorata | 0.0 | 0.3 | 2.5 | 12.7 | 73.8 | 92.7 | 17.4 | 2.2 | 199.4 | 30.1 |
| 21 | Angelica gigas | 0.2 | 2.9 | 9.0 | 7.5 | 5.0 | 1.8 | 0.5 | 19.3 | 27.0 | 26.6 |
| 22 | Sanguisorba obtusa | 1.5 | 16.5 | 109.5 | 363.8 | 601.5 | 3770.4 | 3461.7 | 0.2 | 8324.8 | 31.4 |
| 23 | Rudbeckia maxima | 0.1 | 2.8 | 59.1 | 83.7 | 29.8 | 4.0 | 0.7 | 3.4 | 180.1 | 26.7 |
| 24 | *Paeonia* 'Lovebirds' | 0.1 | 0.7 | 7.2 | 19.4 | 81.8 | 1324.6 | 483.2 | NA | 1917.0 | 31.4 |
| 25 | Amsonia tabernaemontana | 0.6 | 0.6 | 5.9 | 11.0 | 11.0 | 12.8 | 7.0 | 0.5 | 48.9 | 29.0 |
| 26 | Galium odoratum | 0.0 | 1.1 | 10.4 | 127.4 | 116.9 | 5.1 | 0.7 | 1.3 | 261.7 | 27.9 |
| 27 | Laurus nobilis | 0.5 | 7.6 | 9.8 | 5.2 | 4.5 | 3.8 | 0.2 | 7.5 | 31.6 | 26.1 |
| 29 | Geranium 'Brookside' | 0.3 | 2.4 | 3.6 | 20.8 | 88.0 | 451.0 | 537.6 | 0.1 | 1103.6 | 31.7 |
| 31 | Cymbopogon citratus | 0.0 | 0.0 | 0.0 | 10.6 | 12.0 | 16.1 | 4.8 | 2.2 | 43.4 | 29.7 |
| 32 | Cotinus coggygria 'Royal Purple' | 1.4 | 0.4 | 0.8 | 4.2 | 294.3 | 173.1 | 7.5 | 1.1 | 481.8 | 29.7 |
| 33 | Dicksonia antartica | 0.0 | 0.0 | 0.0 | 0.7 | 1.4 | 2.2 | 1.5 | NA | 5.8 | 30.6 |
| 34 | Costus barbatus | 0.0 | 0.3 | 0.7 | 6.1 | 50.4 | 247.4 | 127.9 | 0.2 | 432.9 | 31.3 |
| 35 | Lithachne pauciflora | 5.8 | 4.2 | 1.1 | 3.3 | 7.3 | 2.2 | 1.1 | 8.5 | 24.9 | 26.0 |
| 36 | Piper betle | 0.0 | 0.0 | 0.5 | 1.8 | 11.6 | 78.7 | 61.7 | 2.2 | 154.3 | 31.6 |
| 37 | Dichorisandra thyrsiflora | 0.0 | 0.0 | 0.3 | 0.7 | 2.6 | 3.8 | 10.4 | 3.8 | 17.9 | 31.6 |
| 38 | Mauritiella armata | 0.1 | 0.2 | 0.6 | 1.3 | 14.7 | 163.8 | 209.4 | 0.0 | 390.1 | 32.0 |
| 39 | Brunfelsia pilosa | 0.4 | 1.4 | 2.8 | 3.7 | 15.0 | 6.7 | 2.6 | 0.0 | 32.6 | 28.8 |
| 41 | Bocconia frutescens | 0.1 | 0.4 | 1.5 | 3.7 | 6.1 | 2.4 | 1.1 | 0.4 | 15.4 | 28.5 |
| 42 | Oreopanax capitatus | 0.0 | 0.4 | 8.4 | 83.6 | 151.9 | 542.9 | 100.7 | 0.2 | 887.8 | 30.4 |
| 43 | Chusquea liebmannii | 4.6 | 5.5 | 5.3 | 13.2 | 12.2 | 9.8 | 7.2 | 0.2 | 57.8 | 27.8 |
| 44 | Hevea brasiliensis | 0.3 | 0.1 | 0.9 | 10.5 | 69.3 | 189.3 | 63.5 | 0.0 | 333.8 | 30.8 |
| 45 | Genipa americana | 0.0 | 1.5 | 1.8 | 13.8 | 279.0 | 503.1 | 219.9 | 0.0 | 1019.1 | 30.8 |
| 46 | Elegia capensis | 0.0 | 0.1 | 0.8 | 8.1 | 466.1 | 501.7 | 1.4 | 0.0 | 978.1 | 30.0 |
| 47 | Macleania insignis | 0.1 | 0.5 | 0.9 | 6.9 | 110.2 | 301.3 | 117.0 | 0.0 | 536.9 | 31.0 |
| 48 | Coffea arabica | 0.0 | 0.0 | 0.0 | 2.2 | 209.5 | 146.7 | 11.1 | 0.0 | 369.5 | 29.9 |
| 50 | Spathoglottis plicata | 0.0 | 0.1 | 0.4 | 1.2 | 4.6 | 6.9 | 3.5 | 1.2 | 16.7 | 30.4 |
| 51 | Vanda tricolor var. planilabris | 0.2 | 0.9 | 3.0 | 12.0 | 13.8 | 12.2 | 3.2 | 0.4 | 45.4 | 28.9 |
| 52 | Metasequoia glyptostroboides | 0.0 | 0.6 | 1.2 | 1.0 | 1.9 | 1.2 | 0.4 | NA | 6.2 | 28.0 |
| 53 | Picea orientalis | 0.1 | 0.9 | 1.6 | 2.9 | 9.7 | 6.2 | 1.3 | 13.5 | 22.7 | 29.0 |
| 54 | Phyllostachys nigra | 2.1 | 6.4 | 27.3 | 86.5 | 260.3 | 84.3 | 25.1 | 0.4 | 492.1 | 28.9 |
| 55 | Ginkgo biloba | 0.0 | 0.9 | 2.2 | 7.5 | 2.5 | 1.0 | 0.2 | 20.4 | 14.2 | 27.1 |
| 56 | Liquidambar styraciflua | 0.0 | 0.5 | 2.7 | 21.2 | 280.6 | 563.6 | 209.7 | 0.1 | 1078.3 | 30.8 |
| 57 | Nyssa sylvatica | 0.1 | 1.3 | 4.0 | 29.5 | 239.5 | 33.3 | 1.0 | 0.1 | 308.8 | 29.0 |
| 58 | Taxodium distichum | 0.0 | 0.3 | 0.5 | 1.3 | 1.1 | 0.8 | 1.3 | NA | 5.3 | 29.1 |
| 59 | *Abies homolepis^a^* | **0** | **0.05** | **0.09** | **0.24** | **0.21** | **0.16** | **0.24** | NA | NA | **29.1** |
| 60 | Cercidiphyllum japonicum | 0.1 | 1.2 | 3.4 | 11.7 | 101.7 | 103.8 | 3.5 | 0.9 | 225.3 | 29.8 |
| 61 | Indocalamus tessellatus | 0.2 | 2.9 | 18.5 | 36.0 | 38.0 | 9.6 | 6.4 | NA | 111.5 | 27.9 |
| 62 | Rhapis humilis | 0.0 | 0.8 | 0.4 | 2.1 | 39.1 | 408.1 | 229.9 | NA | 680.4 | 31.5 |
| 63 | Attalea oleveira | 0.0 | 0.2 | 0.3 | 0.6 | 0.4 | 5.3 | 6.7 | 0.3 | 13.4 | 31.5 |
| 64 | Phyllostachys aureosulcata | 0.4 | 0.7 | 3.2 | 10.7 | 99.6 | 42.9 | 6.4 | 0.1 | 164.0 | 29.4 |
| 65 | Chamaedorea pochutlensis | 0.1 | 0.4 | 1.1 | 11.8 | 37.3 | 24.7 | 3.2 | 2.1 | 78.6 | 29.4 |
| 66 | Bougainvillea 'Tahitian Dawn' | 0.0 | 0.1 | 0.7 | 1.8 | 12.5 | 3.1 | 0.5 | 1.0 | 18.6 | 29.1 |
| 67 | Sabal etonia | 0.0 | 0.2 | 0.5 | 1.2 | 2.0 | 4.4 | 12.5 | 12.5 | 20.9 | 31.5 |
| 68 | Prosopis glandulosa | 0.0 | 0.4 | 2.7 | 3.7 | 4.6 | 1.9 | 0.0 | 5.9 | 13.3 | 27.7 |
| 69 | Simmondsia chinensis | 0.0 | 0.4 | 1.3 | 6.2 | 7.8 | 9.2 | 0.9 | 48.8 | 25.8 | 29.1 |
| 70 | Alcantarea imperialis | 0.3 | 0.6 | 0.8 | 0.8 | 1.2 | 0.7 | 0.4 | 5.8 | 4.8 | 27.3 |
| 71 | Oscularia deltoides | 0.0 | 2.0 | 14.0 | 9.6 | 46.4 | 2390.0 | 1209.9 | NA | 3671.8 | 31.6 |
| 72 | Crassula muscosa | 0.0 | 0.0 | 0.3 | 5.2 | 8.5 | 24.3 | 10.4 | NA | 48.6 | 30.6 |
| 74 | Leymus arenarius | 0.1 | 0.6 | 3.1 | 7.8 | 65.9 | 89.0 | 38.4 | 0.0 | 204.8 | 30.5 |
| 75 | Chusquea virgata | 0.1 | 0.4 | 1.4 | 5.8 | 13.7 | 15.8 | 5.7 | NA | 42.8 | 29.8 |
| 76 | Ephedra gerardiana | 0.2 | 1.0 | 3.2 | 20.1 | 96.5 | 30.3 | 0.8 | 0.1 | 152.0 | 29.0 |
| 77 | Brunfelsia pauciflora | 0.0 | 5.5 | 6.7 | 7.4 | 35.0 | 180.5 | 227.5 | 0.0 | 462.6 | 31.6 |
| 79 | Alsophila firma | 0.0 | 0.0 | 0.5 | 7.8 | 7.3 | 2.1 | 1.8 | NA | 19.5 | 28.7 |
| 80 | Zeugites americana | 2.5 | 2.7 | 2.7 | 9.3 | 11.3 | 12.1 | 16.8 | 0.3 | 57.4 | 29.5 |
| 81 | Pinus parviflora | 0.2 | 1.5 | 1.8 | 2.5 | 7.3 | 6.3 | 2.2 | 1.1 | 21.8 | 28.9 |
| 82 | Crassula ovata | 0.0 | 0.3 | 7.1 | 9.9 | 127.0 | 573.9 | 16.4 | 0.0 | 734.6 | 30.6 |
| 83 | Ilex americana | 0.2 | 1.1 | 4.2 | 93.8 | 770.1 | 119.5 | 14.0 | 0.0 | 1002.9 | 29.1 |
| 84 | Alluaudia humbertii | 0.0 | 0.0 | 0.8 | 9.4 | 85.8 | 12.0 | 3.2 | 0.1 | 111.1 | 29.1 |

1. The values for this species is only for *n*-alkane percentage in total *n*-alkanes, due to lack of mass data.
