# Supplementary material for: Major Evolutionary Trends in Hydrogen Isotope Fractionation of Vascular Plant Leaf Waxes
Source: PLoS One. 2014 Nov 17;9(11):e112610. doi: 10.1371/journal.pone.0112610 (PMC4234459; doi:10.1371/journal.pone.0112610)
Supplement: Table S4 — The leaf lipid abundances for each n-alkanoic acid lipid for plant samples collected from the New York Botanic Garden. The unit for single lipids and sums is µg/g d.w. leaf. ACL is the average chain length (ACL = ∑n*Cn/sum, where n is carbon number 24, 26, 28, 30, and 32, Cn is lipid with n carbon number and sum is total mass of C24–C32 n-acids). (DOC) [file pone.0112610.s011.doc]

**Table S4.** The leaf lipid abundances for each *n*-alkanoic acid lipid for plant samples collected from the New York Botanic Garden. The unit for single lipids and sums is µg/g d.w. leaf. ACL is the average chain length (ACL= ∑n*Cn /sum, where n is carbon number 24, 26, 28, 30, and 32, Cn is lipid with n carbon number and sum is total mass of C24-C32 n-acids).

| **ID** | **Species** | **C20** | **C22** | **C24** | **C26** | **C28** | **C30** | **C32** | **sum- C20-32** | **Total**  **C24-32** | **ACL C24-32** |
| --- | --- | --- | --- | --- | --- | --- | --- | --- | --- | --- | --- |
| 1 | Hosta plantaginea 'Aphrodite' | 17.4 | 20.0 | 28.4 | 42.5 | 42.6 | 39.9 | 35.5 | 226.4 | 153.5 | 27.2 |
| 2 | Polygonatum odoratum var. pluriflorum 'Variegatum' | 6.9 | 67.9 | 82.9 | 162.7 | 100.5 | 0.0 | 0.0 | 420.9 | 346.2 | 26.1 |
| 3 | Phalaris arundinacea var. picta 'Picta' | 51.8 | 24.2 | 26.8 | 179.1 | 121.0 | 76.7 | 19.4 | 498.9 | 403.5 | 27.2 |
| 4 | Miscanthus sinensis 'Gracillimus' | 26.1 | 19.4 | 27.3 | 18.4 | 71.1 | 88.0 | 0.0 | 250.3 | 204.8 | 28.1 |
| 5 | Stipa calamagrostis | 21.0 | 27.1 | 69.9 | 41.6 | 59.7 | 33.2 | 37.4 | 289.9 | 204.5 | 26.6 |
| 6 | Allium christophii | 15.5 | 44.3 | 24.4 | 44.8 | 87.0 | 31.6 | 14.6 | 262.2 | 187.9 | 27.3 |
| 7 | Rodgersia podophylla | 13.7 | 38.0 | 56.0 | 25.1 | 19.1 | 15.9 | 3.9 | 171.6 | 116.1 | 25.9 |
| 8 | Dicentra spectabilis | 12.2 | 109.7 | 145.4 | 112.1 | 673.1 | 807.4 | 0.0 | 1859.8 | NA | NA |
| 9 | Convallaria majalis | 22.6 | 16.5 | 25.7 | 24.6 | 76.3 | 64.6 | 0.0 | 230.3 | 191.2 | 27.9 |
| 10 | Lilium 'Pink Twinkle' asiatic hybrid lilly | 7.9 | 9.0 | 27.0 | 33.8 | 70.4 | 134.9 | 52.4 | 335.3 | 266.0 | 28.4 |
| 11 | Matteuccia struthiopteris | 25.0 | 9.8 | 30.1 | 20.9 | 7.4 | 1.8 | 0.0 | 95.0 | 60.1 | 25.4 |
| 12 | Hakonechloa macra 'Aureola' | 37.0 | 30.2 | 43.5 | 23.9 | 2.2 | 0.0 | 0.0 | 136.8 | 69.6 | 24.8 |
| 13 | Asarum europaeum | 7.3 | 3.3 | 6.5 | 15.6 | 6.0 | 12.1 | 17.2 | 67.9 | 40.2 | 27.2 |
| 14 | Adiantum pedatum | 15.4 | 24.6 | 39.4 | 19.4 | 65.4 | 115.7 | 0.0 | 279.8 | 239.8 | 28.1 |
| 15 | Carex oshimensis | 28.4 | 44.4 | 65.0 | 60.7 | 27.3 | 19.1 | 14.0 | 258.9 | 172.2 | 26.0 |
| 16 | Nicotiana mutabilis | 6.9 | 13.0 | 18.5 | 9.7 | 3.5 | 3.5 | 3.4 | 58.6 | 35.3 | 25.6 |
| 17 | Iris sp. | 0.1 | 0.3 | 1.1 | 3.6 | 4.3 | 2.4 | 0.2 | 12.0 | 11.4 | 27.4 |
| 18 | Baptisia australis | 42.9 | 6.8 | 5.0 | 41.7 | 40.1 | 14.4 | 6.6 | 157.5 | 101.3 | 27.3 |
| 19 | Salvia transsylvanica | 4.1 | 2.4 | 6.8 | 11.9 | 41.5 | 39.3 | 3.0 | 109.0 | 99.5 | 28.3 |
| 20 | Euphorbia collorata | 34.9 | 32.0 | 19.3 | 32.4 | 142.4 | 120.6 | 49.5 | 431.2 | 314.7 | 28.3 |
| 21 | Angelica gigas | 2.6 | 16.5 | 96.5 | 187.9 | 131.5 | 69.9 | 15.3 | 520.3 | 485.9 | 26.7 |
| 22 | Sanguisorba obtusa | 43.7 | 46.0 | 325.6 | 516.3 | 306.0 | 63.9 | 69.2 | 1370.6 | 1211.7 | 26.2 |
| 23 | Rudbeckia maxima | 76.2 | 36.5 | 34.5 | 153.8 | 216.0 | 82.8 | 11.4 | 611.2 | 487.1 | 27.4 |
| 25 | Amsonia tabernaemontana | 1.8 | 1.2 | 2.1 | 4.8 | 8.6 | 5.1 | 0.0 | 23.7 | 20.6 | 27.6 |
| 26 | Galium odoratum | 4.3 | 8.8 | 22.7 | 62.4 | 135.7 | 108.3 | 3.1 | 345.3 | 329.2 | 28.0 |
| 27 | Laurus nobilis | 12.5 | 7.0 | 79.2 | 70.1 | 39.6 | 27.5 | 1.4 | 237.4 | 216.5 | 26.1 |
| 29 | Geranium 'Brookside' | 3.1 | 7.8 | 15.5 | 38.2 | 23.1 | 20.7 | 9.2 | 117.7 | 97.5 | 27.0 |
| 31 | Cymbopogon citratus | 20.9 | 38.2 | 28.2 | 6.1 | 4.3 | 0.0 | 0.0 | 97.7 | 38.6 | 24.8 |
| 32 | Cotinus coggygria 'Royal Purple' | 73.0 | 25.4 | 110.0 | 30.0 | 46.2 | 186.8 | 37.6 | 509.0 | 373.0 | 27.7 |
| 34 | Costus barbatus | 1.2 | 18.2 | 7.1 | 6.5 | 41.2 | 17.2 | 8.0 | 99.4 | 71.9 | 27.9 |
| 35 | Lithachne pauciflora | 4.6 | 22.6 | 18.3 | 25.7 | 28.1 | 83.1 | 29.1 | 211.5 | 155.2 | 28.3 |
| 36 | Piper betle | 6.7 | 14.4 | 59.1 | 138.5 | 76.7 | 50.3 | 1.3 | 346.9 | 324.6 | 26.7 |
| 37 | Dichorisandra thyrsiflora | 11.2 | 9.8 | 17.5 | 11.3 | 9.4 | 5.1 | 3.8 | 68.2 | 43.4 | 26.1 |
| 38 | Mauritiella armata | 0.1 | 0.1 | 0.2 | 0.2 | 0.2 | 0.6 | 0.0 | 1.4 | 1.2 | 28.1 |
| 39 | Brunfelsia pilosa | 0.4 | 0.4 | 0.2 | 0.2 | 0.2 | 0.2 | 0.0 | 1.6 | 0.8 | 26.9 |
| 41 | Bocconia frutescens | 0.5 | 0.6 | 0.6 | 0.9 | 1.7 | 1.7 | 0.0 | 6.1 | 5.0 | 27.9 |
| 42 | Oreopanax capitatus | 2.5 | 10.9 | 20.4 | 14.9 | 58.7 | 45.5 | 28.7 | 181.6 | 139.5 | 27.9 |
| 43 | Chusquea liebmannii | 0.4 | 0.5 | 1.0 | 1.8 | 2.3 | 2.7 | 1.1 | 9.7 | 7.8 | 27.7 |
| 44 | Hevea brasiliensis | 0.0 | 0.0 | 0.1 | 0.2 | 1.6 | 1.2 | 0.0 | 3.1 | 3.1 | 28.5 |
| 45 | Genipa americana | 0.4 | 0.2 | 0.1 | 0.2 | 0.7 | 0.5 | 0.0 | 2.1 | 1.5 | 28.0 |
| 46 | Elegia capensis | 10.5 | 6.7 | 10.0 | 8.9 | 0.0 | 0.0 | 0.0 | 36.1 | 18.9 | 24.9 |
| 47 | Macleania insignis | 0.1 | 0.2 | 0.5 | 0.8 | 0.0 | 0.0 | 0.0 | 1.6 | 1.3 | 25.2 |
| 48 | Coffea arabica | 0.1 | 0.1 | 0.1 | 0.2 | 0.0 | 0.0 | 0.0 | 0.4 | 0.3 | 25.4 |
| 49 | Selaginella umbrosa | 0.5 | 0.8 | 3.2 | 4.4 | 2.7 | 0.9 | 0.3 | 12.9 | 11.2 | 26.2 |
| 50 | Spathoglottis plicata | 2.2 | 1.1 | 3.9 | 2.9 | 3.4 | 6.8 | 0.0 | 20.2 | 16.9 | 27.5 |
| 51 | Vanda tricolor var. planilabris | 2.9 | 0.9 | 1.5 | 1.2 | 3.6 | 4.3 | 3.7 | 18.0 | 10.5 | 28.0 |
| 53 | Picea orientalis | 47.4 | 32.5 | 38.8 | 12.7 | 12.0 | 84.7 | 77.6 | 305.8 | 148.2 | 27.9 |
| 54 | Phyllostachys nigra | 2.6 | 8.8 | 26.4 | 45.7 | 47.8 | 65.7 | 21.8 | 218.8 | 185.6 | 27.6 |
| 55 | Ginkgo biloba | 4.3 | 17.8 | 102.5 | 63.2 | 74.8 | 23.2 | 3.3 | 289.1 | 263.7 | 26.1 |
| 56 | Liquidambar styraciflua | 3.7 | 5.4 | 17.0 | 15.3 | 19.7 | 18.2 | 12.5 | 91.8 | 70.2 | 27.1 |
| 57 | Nyssa sylvatica | 3.3 | 2.9 | 16.5 | 12.5 | 4.0 | 1.6 | 2.3 | 43.2 | 34.7 | 25.5 |
| 60 | Cercidiphyllum japonicum | 4.1 | 6.7 | 45.4 | 16.2 | 44.3 | 63.6 | 18.4 | 198.7 | 169.5 | 27.5 |
| 63 | Attalea oleveira | 0.3 | 0.6 | 1.5 | 0.3 | 1.0 | 0.7 | 0.0 | 4.4 | 3.5 | 26.5 |
| 64 | Phyllostachys aureosulcata | 0.0 | 0.8 | 1.5 | 2.8 | 3.8 | 4.3 | 0.0 | 13.3 | 12.5 | 27.8 |
| 65 | Chamaedorea pochutlensis | 6.8 | 14.1 | 17.6 | 10.4 | 33.7 | 54.6 | 29.1 | 166.3 | 116.3 | 28.2 |
| 66 | Bougainvillea 'Tahitian Dawn' | 0.0 | 0.3 | 1.7 | 4.4 | 7.5 | 4.4 | 1.1 | 19.5 | 18.0 | 27.6 |
| 67 | Sabal etonia | 3.5 | 20.2 | 35.1 | 8.3 | 117.7 | 17.0 | 58.4 | 260.2 | 178.1 | 27.3 |
| 68 | Prosopis glandulosa | 35.9 | 19.9 | 8.1 | 5.8 | 8.1 | 1.2 | 0.0 | 79.1 | 23.3 | 26.2 |
| 69 | Simmondsia chinensis | 3.9 | 59.7 | 262.8 | 233.8 | 616.9 | 61.7 | 18.8 | 1257.6 | 1175.2 | 26.8 |
| 70 | Alcantarea imperialis | 1.4 | 3.5 | 3.3 | 3.7 | 5.9 | 8.5 | 1.9 | 28.2 | 21.5 | 27.8 |
| 74 | Leymus arenarius | 0.5 | 0.7 | 0.7 | 0.7 | 1.1 | 1.1 | 0.0 | 4.8 | 3.6 | 27.4 |
| 76 | Ephedra gerardiana | 0.6 | 1.7 | 1.8 | 0.9 | 3.8 | 6.6 | 1.4 | 16.8 | 13.2 | 28.3 |
| 77 | Brunfelsia pauciflora | 0.4 | 0.6 | 0.6 | 0.5 | 0.5 | 0.8 | 1.0 | 4.3 | 2.4 | 27.3 |
| 80 | Zeugites americana | 2.4 | 0.8 | 2.3 | 3.4 | 2.7 | 2.4 | 1.4 | 15.2 | 10.8 | 26.9 |
| 81 | Pinus parviflora | 13.9 | 2.2 | 2.0 | 1.2 | 1.1 | 3.1 | 0.0 | 23.5 | 7.4 | 27.4 |
| 82 | Crassula ovata | 0.8 | 0.2 | 0.2 | 0.2 | 0.8 | 0.0 | 0.0 | 2.3 | 1.3 | 26.9 |
| 83 | Ilex americana | 0.2 | 0.3 | 1.2 | 1.3 | 3.5 | 4.3 | 0.5 | 11.2 | 10.2 | 28.1 |
| 84 | Alluaudia humbertii | 3.2 | 0.5 | 0.6 | 1.1 | 1.8 | 0.7 | 0.4 | 8.3 | 4.2 | 27.3 |
