# Supplementary material for: Major Evolutionary Trends in Hydrogen Isotope Fractionation of Vascular Plant Leaf Waxes
Source: PLoS One. 2014 Nov 17;9(11):e112610. doi: 10.1371/journal.pone.0112610 (PMC4234459; doi:10.1371/journal.pone.0112610)
Supplement: Table S5 — The centered and VAST-scaled ε*wax-xylem values for the 7 leaf lipids for each plant species. The detailed description for the scaling and combination is described in “Methods”. (DOC) [file pone.0112610.s012.doc]

**Table S5.** The centered and VAST-scaled ε*_wax-xylem_ values for the 7 leaf lipids for each plant species. The detailed description for the scaling and combination is described in “Methods”.

| **ID** | **Scientific Name** | **C24_vast** | **C26_vast** | **C28_vast** | **C30_vast** | **C27_vast** | **C29_vast** | **C31_vast** |
| --- | --- | --- | --- | --- | --- | --- | --- | --- |
| sp1 | Selaginella moellendorffii | 26 | 35 | NaN | NaN | 39 | 43 | NaN |
| sp2 | Selaginella huehuetenangensis | 34 | 53 | 51 | NaN | 37 | 39 | 52 |
| sp3 | Selaginella schiedeana | 41 | 39 | 52 | 56 | 28 | 56 | NaN |
| sp4 | Selaginella kraussiana | -23 | -24 | -11 | -20 | -42 | -6 | NaN |
| sp5 | Huperzia squarrosa | 37 | 40 | 72 | NaN | 30 | 43 | NaN |
| sp6 | Huperzia sp. | 42 | 52 | 89 | NaN | 58 | 63 | NaN |
| sp7 | Selaginella mosorongensis | 30 | 33 | 36 | 0 | 18 | -4 | -13 |
| sp8 | Selaginella mickelii | 34 | 44 | 55 | 88 | 30 | 34 | 45 |
| sp9 | Selaginella galeottii | NaN | NaN | NaN | NaN | 54 | NaN | NaN |
| sp10 | Selaginella brauniii | -9 | -12 | -3 | NaN | 17 | 32 | NaN |
| sp11 | Dicksonia antartica | -13 | 2 | 17 | 24 | NaN | 14 | -7 |
| sp12 | Alsophila firma | 48 | 51 | 22 | 49 | 39 | 51 | 42 |
| sp13 | Matteuccia struthiopteris | 24 | 24 | 28 | 42 | 9 | 23 | -10 |
| sp14 | Adiantum pedatum | 39 | 37 | 52 | NaN | 22 | -36 | NaN |
| sp15 | Marattia attenuata | 12 | 0 | -36 | 12 | NaN | NaN | NaN |
| sp16 | Dennstaedtia globulifera | 27 | 20 | 30 | 21 | 45 | 35 | NaN |
| sp17 | Asplenium formosanum | -15 | -20 | -12 | -8 | NaN | -12 | -6 |
| sp18 | Thelypteris sp. | 36 | 46 | 48 | 39 | 38 | 39 | 27 |
| sp19 | Blechnum glandulosum | 20 | 26 | 37 | 29 | 32 | 35 | NaN |
| sp20 | Arachniodes amabilis | 4 | 6 | 12 | 3 | 17 | 20 | 2 |
| sp21 | Arthropteris articulata | 14 | 49 | 36 | 49 | NaN | 18 | 13 |
| sp22 | Bolbitis portoricensis | 27 | 26 | 38 | 27 | NaN | NaN | 9 |
| sp23 | Campyloneurum angustifolium | 34 | 38 | 37 | 43 | 36 | 34 | 31 |
| sp24 | Tectaria zeylanica | 8 | 9 | 16 | 11 | 9 | 16 | 6 |
| sp25 | Osmunda regalis | 19 | 7 | 14 | 17 | -13 | -5 | 12 |
| sp26 | Osmundastrum cinnamomeum | 25 | 26 | 30 | 19 | -7 | 8 | 27 |
| sp27 | Sphaeropteris horrida | -1 | 55 | 60 | 39 | 16 | 24 | NaN |
| sp28 | Davallia fejeensis 'Plumosa' | 28 | 33 | 40 | 38 | 8 | 25 | 22 |
| sp29 | Davallia trichomanoides | 27 | 29 | 57 | 43 | NaN | 32 | 14 |
| sp30 | Taxodium distichum | 21 | 15 | 25 | NaN | -8 | 2 | 17 |
| sp31 | Abies homolepis | NaN | -2 | 15 | NaN | -7 | -1 | 31 |
| sp32 | Metasequoia glyptostroboides | -3 | 5 | 24 | NaN | 0 | 4 | 17 |
| sp33 | Picea orientalis | -8 | 10 | 25 | 21 | NaN | NaN | NaN |
| sp34 | Ginkgo biloba | -36 | 13 | 11 | 23 | 0 | 3 | 18 |
| sp35 | Ephedra gerardiana | 26 | 36 | 24 | NaN | 12 | 19 | 11 |
| sp36 | Pinus parviflora | -20 | -16 | -15 | -10 | 0 | -14 | -9 |
| sp37 | Bougainvillea 'Tahitian Dawn' | 8 | 12 | 12 | 8 | 10 | 6 | 2 |
| sp38 | Amsonia tabernaemontana | -46 | -53 | -49 | -38 | -49 | -43 | -22 |
| sp39 | Galium odoratum | 11 | 14 | 19 | 8 | NaN | 21 | 6 |
| sp40 | Salvia transsylvanica | 20 | 35 | 23 | 32 | 11 | 8 | 20 |
| sp41 | Nicotiana mutabilis | 10 | -8 | -24 | -10 | -3 | 8 | -3 |
| sp42 | Rudbeckia maxima | -6 | -11 | 5 | 17 | -23 | -4 | NaN |
| sp43 | Euphorbia collorata | 9 | 15 | 4 | 11 | -3 | -1 | 1 |
| sp44 | Baptisia australis | -6 | -11 | 8 | 15 | NaN | NaN | 16 |
| sp45 | Sanguisorba obtusa | -3 | -15 | -5 | 2 | -18 | -7 | NaN |
| sp46 | Geranium 'Brookside' | 30 | 54 | 22 | 26 | 10 | -16 | -20 |
| sp47 | Paeonia 'Lovebirds' | 20 | 19 | 35 | 39 | NaN | -10 | NaN |
| sp48 | Dicentra spectabilis | 27 | 36 | 26 | 31 | 19 | 16 | -1 |
| sp49 | Asarum europaeum | -16 | -18 | -10 | -23 | NaN | -11 | -28 |
| sp50 | Rodgersia podophylla | 3 | -3 | -13 | -11 | NaN | 30 | 0 |
| sp51 | Genipa americana | -61 | -51 | -70 | -60 | -75 | -84 | -19 |
| sp52 | Nyssa sylvatica | 45 | 48 | 77 | 50 | 37 | 45 | 39 |
| sp53 | Hevea brasiliensis | NaN | NaN | NaN | NaN | NaN | NaN | NaN |
| sp54 | Cercidiphyllum japonicum | 24 | 27 | 13 | -60 | 28 | -11 | 38 |
| sp55 | Liquidambar styraciflua | 53 | 68 | 62 | 51 | NaN | 45 | 24 |
| sp56 | Laurus nobilis | 4 | 13 | NaN | NaN | 66 | 10 | 4 |
| sp57 | Coffea arabica | 17 | 10 | 17 | -6 | NaN | 12 | 13 |
| sp58 | Brunfelsia pilosa | 32 | 20 | -3 | 21 | NaN | 9 | 4 |
| sp59 | Ilex americana | 45 | 59 | NaN | 22 | 13 | 16 | 27 |
| sp60 | Prosopis glandulosa | -17 | -21 | -5 | 12 | 34 | 1 | -1 |
| sp61 | Cotinus coggygria 'Royal Purple' | 31 | 39 | 45 | 30 | -28 | -30 | -2 |
| sp62 | Bocconia frutescens | 10 | 25 | 23 | 34 | NaN | 24 | 27 |
| sp63 | Simmondsia chinensis | -11 | -22 | -19 | -13 | -8 | 0 | 24 |
| sp64 | Angelica gigas | -12 | -12 | -2 | 12 | -7 | 3 | -1 |
| sp65 | Oreopanax capitatus | 18 | 24 | 29 | 30 | 9 | 24 | 44 |
| sp66 | Macleania insignis | -9 | -14 | -14 | 2 | -3 | 16 | 19 |
| sp67 | Piper betle | NaN | NaN | NaN | NaN | NaN | NaN | NaN |
| sp68 | Brunfelsia pauciflora | 34 | 13 | NaN | -27 | NaN | 30 | -3 |
| sp69 | Oscularia deltoides | NaN | NaN | NaN | NaN | NaN | 36 | 4 |
| sp70 | Alluaudia humbertii | NaN | NaN | NaN | NaN | 32 | 0 | 5 |
| sp71 | Crassula ovata | 17 | 27 | 6 | 28 | NaN | 40 | 22 |
| sp72 | Crassula muscosa | NaN | NaN | NaN | NaN | NaN | NaN | NaN |
| sp73 | Dichorisandra thyrsiflora | -24 | -28 | -35 | -39 | -14 | -25 | -20 |
| sp74 | Costus barbatus | 1 | -13 | -6 | 0 | NaN | 12 | -2 |
| sp75 | Iris sp. | -2 | -20 | -24 | -26 | -16 | -9 | -11 |
| sp76 | Spathoglottis plicata | -4 | -17 | -17 | -14 | -5 | -12 | -25 |
| sp77 | Vanda tricolor var. planilabris | -14 | -23 | -32 | -20 | -32 | -32 | -31 |
| sp78 | Hosta plantaginea 'Aphrodite' | -17 | -34 | -42 | -44 | -32 | -28 | -10 |
| sp79 | Convallaria majalis | 9 | 0 | -4 | 1 | -27 | -19 | NaN |
| sp80 | Lilium 'Pink Twinkle' asiatic hybrid lilly | -22 | -42 | -45 | -34 | 34 | -2 | NaN |
| sp81 | Allium christophii | -34 | -36 | -54 | -34 | 4 | -32 | -55 |
| sp82 | Polygonatum odoratum var. pluriflorum 'Variegatum' | -19 | -24 | -10 | -1 | -15 | -7 | 8 |
| sp83 | Chamaedorea pochutlensis | -30 | -33 | -39 | -27 | -33 | -38 | -27 |
| sp84 | Rhapis humilis | -14 | -31 | -33 | -30 | NaN | 1 | -31 |
| sp85 | Attalea oleveira | -45 | -41 | -47 | -31 | 4 | 10 | 25 |
| sp86 | Mauritiella armata | NaN | NaN | NaN | NaN | NaN | NaN | NaN |
| sp87 | Sabal etonia | NaN | NaN | NaN | NaN | NaN | NaN | NaN |
| sp88 | Phalaris arundinacea var. picta 'Picta' | -47 | -58 | -45 | -40 | NaN | NaN | NaN |
| sp89 | Miscanthus sinensis 'Gracillimus' | -25 | 0 | -30 | -24 | -26 | -23 | -12 |
| sp90 | Stipa calamagrostis | -55 | -56 | -58 | -60 | -50 | -88 | -71 |
| sp91 | Hakonechloa macra 'Aureola' | -48 | -54 | -71 | -44 | -12 | -40 | -45 |
| sp92 | Carex oshimensis | -49 | -43 | -35 | -20 | -6 | -36 | -26 |
| sp93 | Cymbopogon citratus | -14 | -20 | -10 | 6 | -21 | -2 | 3 |
| sp94 | Lithachne pauciflora | -52 | -68 | -64 | -60 | -16 | -33 | -34 |
| sp95 | Chusquea liebmannii | -71 | -82 | -76 | -58 | -60 | -70 | -40 |
| sp96 | Phyllostachys nigra | -26 | -51 | -43 | -25 | -59 | -60 | -26 |
| sp97 | Indocalamus tessellatus | -19 | -37 | -68 | -59 | -53 | -48 | NaN |
| sp98 | Phyllostachys aureosulcata | -46 | -81 | -69 | NaN | -38 | -55 | -41 |
| sp99 | Alcantarea imperialis | -31 | -42 | -45 | -42 | -25 | -33 | 10 |
| sp100 | Leymus arenarius | -67 | -71 | -73 | -64 | NaN | -53 | -50 |
| sp101 | Chusquea virgata | -33 | -68 | -71 | -57 | -26 | -45 | -27 |
| sp102 | Zeugites americana | -36 | -30 | -40 | -42 | -45 | -52 | -51 |
